# A novel metaheuristic optimizer GPSed via artificial intelligence for reliable economic dispatch

**DOI:** 10.1038/s41598-025-06648-9

**Published:** 2025-06-23

**Authors:** Mahmoud Ibrahim Mohamed, Ali M. Yousef, Ahmed A. Hafez

**Affiliations:** Electrical Engineering Department, Assiut University, Assiut, Egypt

**Keywords:** Artificial intelligence, Artificial neural networks, Genetic algorithm, Particle swarm optimization, Teaching–learning-based optimization, Artificial gorilla troops optimization, Economic dispatch, Electrical and electronic engineering, Energy grids and networks

## Abstract

Recently, meta-heuristic optimization algorithms have enhanced resource efficiency, facilitated informed decision-making, and addressed complex problems involving multiple variables and constraints in engineering and science fields. However, numerous handicaps are reported on the performance of a quite number of these optimizers, such as local solution trapping, slow convergence and the requirements for elevated storage and computation capability. This article proposes a novel, simple, and elaborate remedy for the reported deficiencies of meta-heuristic optimizers. This deficiency is accomplished by proposing a hybrid optimizer composed of an ambiguous optimizer and Artificial Intelligence (AI). The performance of the proposed technique is evaluated using four different meta-heuristic optimizers: Genetic Algorithm (GA), Particle Swarm Optimization (PSO), Teaching–Learning-Based Optimization (TLBO), and Artificial Gorilla Troops Optimization (AGTO). These optimizers range from the mature to the recently evolved. These meta-heuristic optimizers validate the proposed solver and confirm its applicability to any meta-heuristic optimization algorithm. Economic Dispatch (ED) of the IEEE 30-bus system is utilized to evaluate the performance of the proposed solver. The comprehensive results demonstrate the superiority, reliability, and adequacy of the proposed technique. It consistently converges to the global optimum solution, achieving the minimum energy cost of the system under concern while requiring the fewest iterations and minimal computational requirements.

## Introduction

Optimization is determining the best and most optimal solution among a set of available solutions to maximize/minimize a function known as the objective function, which is subjected to various constraints specified by the user. Optimization is carried out through an iterative process to improve the potential solutions. Optimization is vital in numerous fields including engineering, computer science, medical science, economics, finance, and many other disciplines. It enhances resource efficiency, supports informed decision-making, and addresses complex problems involving multiple variables and constraints^1,2^. Optimization techniques have been continuously evolving from meta-heuristic optimizers to, most recently, AI-based approaches.

Numerous robust meta-heuristic optimizers are reported in the literature^3–15^, such as GA, PSO, TLBO, and AGTO. GA, PSO, and TLBO are relatively mature optimizers with different degrees of maturity, while AGTO is a more recent candidate. These optimizers vary in the search mechanism and problem handling. Moreover, they differ according to the natural biological/environmental and/or industrial process from which they emerged. For example, GA simulates natural selection by starting with a population of solutions (chromosomes), evaluating their fitness using an objective function, and selecting the best for the next generation. The process continues through iterations with possible mutations until the stopping criterion, like a set iteration count or target quality, is achieved^3,16^. PSO, inspired by the collective behavior of bird flocks and fish schools, treats each solution as a particle with position and velocity. Particles update their state based on personal and neighbor best positions, iteratively converging to the optimal solution, which works effectively for complex, high-dimensional problems^4,8–11^. TLBO, based on the teaching–learning process in classrooms, models a population of students as solutions. The performance of each student is assessed using a fitness function. TLBO operates in two stages: the teacher phase, where knowledge is imparted, and the student phase, where peer interactions enhance learning^12–14^. AGTO mimics the foraging behavior of gorilla troops, balancing exploration and exploitation to converge on optimal solutions. By combining individual and collective intelligence. AGTO is particularly suited for solving complex optimization problems in computer science, engineering, and economics and is effective for multi-objective, dynamic, continuous, and discrete tasks ^5–7,15^.

Meta-heuristic optimizers are widely used for their advantages in handling complex and non-linear problems and achieving high efficiency^17,18^. However, they have drawbacks, such as the risk of getting trapped in local optima and increased computational demands. Typically, these optimizers begin the search process with an initial solution that is either user-provided or generated internally. Since this starting point is random, it introduces challenges like sensitivity, which can affect the performance of the optimizer.

Machine Learning (ML), a key tool in AI, offers a wide range of applications by creating models and methods that can learn efficiently without unnecessary complexity. Through this learning process, it can make decisions autonomously without requiring user intervention or reprogramming. ML mimics the ability of the human mind to learn from experience and make informed decisions^19^. One of the most prominent techniques in the ML field is Artificial Neural Networks (ANN), which is extensively used due to its ability to handle complex operations that are often too challenging for traditional computational methods; they do so in remarkably short periods^18^. A primary application of ANN is prediction, where the system learns from historical data to produce accurate outputs based on simple inputs and parameters^20^. Despite their remarkable capabilities, ANN has certain limitations, such as a lack of robustness. They could be sensitive to changes in input and the issue of local minima, where the network may settle on a suboptimal solution instead of reaching the global optimum. Errors during the learning process will inevitably affect the output quality later^18^.

The deficiencies of meta-heuristic optimizers and AI techniques have been investigated, while hybrid techniques are reported to remedy such limitations. These approaches combine different meta-heuristic optimizers or integrate AI with meta-heuristic methods to harness their strengths, remedy the deficiencies and achieve better results^18^. In Refs.^19–22^, GA is introduced to optimize and correct the initial weights and thresholds of the backpropagation deep neural network. In Ref.^23^, different optimization algorithms (PSO, GA, Biogeography-Based Optimization (BBO), and Grey Wolf Optimization (GWO)) were employed to optimize the parameters of an Adaptive Network-based Fuzzy Inference System (ANFIS). In Ref.^24^, the research presents a comparative analysis of the performance of three ML models—PSO–ANN, GPR, and GA-ANFIS—in predicting the Factor of Safety (FOS) of soil slope stability. Specifically, the study utilizes PSO and GA to optimize the parameters of the ANN and ANFIS models, respectively. Subsequently, the optimized ANN and ANFIS models are employed to predict the FOS of the soil slope, leveraging the optimized parameters to enhance the accuracy of the predictions. In Ref.^25^, the PSO algorithm generates a set of candidate turbine layouts used as input for the ANN. The ANN evaluates these layouts by predicting the power output and providing feedback to the PSO algorithm. This feedback helps guide the PSO toward regions of the search space that are more likely to produce optimal solutions. Ref.^26^ proposes a hybrid approach combining Rank-Gaussian Particle Swarm Optimization (RGPSO) and an ANN. The PSO algorithm optimizes the hyperparameters of the ANN model, which then uses these optimized parameters to predict the surface waviness in wire and arc additive manufacturing components. The predicted waviness serves as feedback for the PSO, allowing it to adjust particle positions and continue searching for improved solutions that minimize surface waviness. In Ref.^27^, the research addresses the Distributed Hybrid Flow Shop Problem (DHFSP) by integrating Q-learning into meta-heuristic local search operations. A random population is generated, and its mass is calculated to represent the default optimal solution. During the iterative process, the optimal solution is continuously updated based on fitness values, following the specific population updating strategies of the employed meta-heuristics. In Ref.^28^, a novel approach is proposed that comprises reinforcement learning with genetic algorithms (RLGA) to enhance the selection of crowdsourced test reports. The reinforcement learning component dynamically adjusts the mutation probability (Pm) and crossover probability (Pc) within the GA framework. In Ref.^29^, Surrogate-Assisted Hybrid Optimization (SAHO) integrates TLBO and Differential Evolution (DE). TLBO emphasizes global exploration, while DE focuses on local exploitation. These algorithms are applied alternately when no improved candidate solution is identified. In Ref.^30^, the study integrates the Q-Learning technique with meta-heuristic algorithms to regulate the balance between the optimization phases, aiming to help determine whether to pursue exploration or exploitation during optimization.

The hybridization between the AI tools and meta-heuristic optimizer is carried out to improve the quality and characteristics of the AI tools, such as hyperparameters of ANN. However, less literature is likely reported regarding using AI to improve the search capabilities of the meta-heuristic optimizers, which acts as a primary objective for our research here in this article.

One of the most significant applications of optimization in electrical engineering is ED. ED is used in this research to examine and validate the proposed technique. Therefore, it is briefly reviewed in this paragraph. ED problem is typically formulated as a constrained optimization problem, where the objective function is defined as the total generation cost. It is optimized to enhance the power system’s generation capabilities while meeting the specified constraints^18^. One major drawback of many optimization methods, particularly in the context of ED, is their reliance on a randomly initial population set within the generation limits of each generator. This approach creates a broad search space, which increases the time required to find an optimal solution. Furthermore, the final solution varies with each run due to the randomness of the starting point, making the results highly sensitive to the number of iterations and population size. Researchers often run the system multiple times using the average outcome to mitigate this variability. However, this approach is time-intensive and laborious, rendering it impractical for critical or time-sensitive applications that require rapid and reliable results, such as ED.

This article presents an innovative new method to overcome the previous disadvantages of optimization methods and benefit from their advantages by integrating the meta-heuristic optimizer and AI in an innovative approach. The proposed optimizer comprises the ANN phase, and the second phase is one of the population-based optimization methods. ANN here is a guide for the other method, guiding it to the beginning of each process with a stable solution very close to the optimal solution. This guideness helps it to reach the optimal solution with high speed and accuracy. The claimed contributions are:

- A new hybrid optimization method is developed by integrating ANN with an optimization method, i.e., GA, PSO, TLBO, and AGTO.
- Applying the proposed optimization techniques to solve the ED problem.
- Carrying out a comprehensive comparison of the proposed hybrid techniques with traditional optimization methods.

The paper is structured as follows: “Proposed AI-GPSed Optimizer” presents the integration of AI with various optimization techniques. Section “Problem description” outlines the ED problem description. Sections “System under concern” and “Study cases” present the system and the cases under study, respectively. The section “Results and Discussions” discusses the results in detail. Section “Comparison and Statistical Performance” provides a summary comparison between the targeted methods. Finally, “Conclusion and future works” summarizes the conclusions and future work.

## Proposed AI-GPSed optimizer

The proposed optimizer is themed AI-GPSed optimizer. This metaphorical terminology is adopted to highlight the role of ANN, one of the AI tools, for guiding the hyperspace search of meta-heuristic optimizers.

**Fig. 1:**
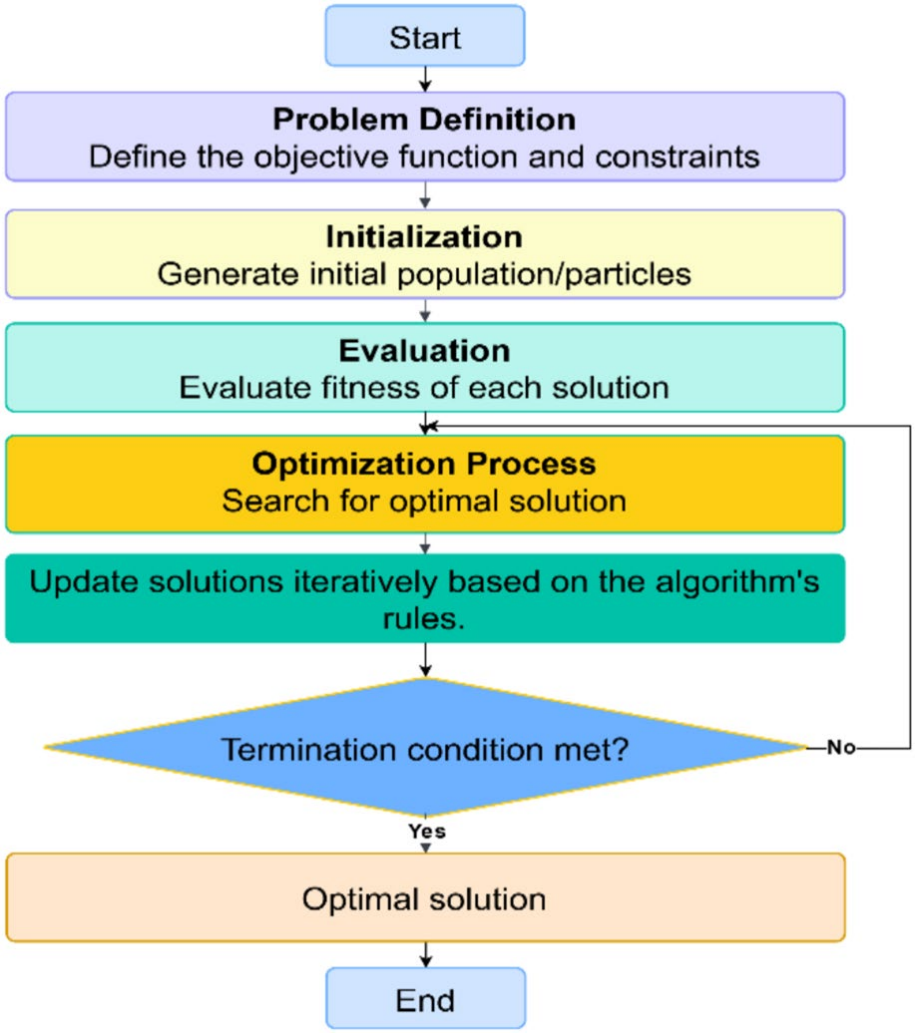
Key steps commonly involved in an optimization process.

Meta-heuristic optimization algorithms generally follow nearly a number of key steps, as illustrated in the flowchart in Fig. 1.

Optimizers require an initial solution to begin the search within the defined search space, Fig. 1, a process referred to as initialization. This initial solution could either be provided by the user or generated internally. In either case, the starting solution typically consists of a random dataset without prior knowledge of the problem. It is reported that the initial solution plays a leading role in the convergence accuracy and/or speed^31,32^. Moreover, the starting solution would affect the execution time. Then, the search space of the meta-heuristic optimizer is usually bounded by constraints, often representing realistic or practical operating conditions. These boundaries tend to increase search complexity, requiring more iterations and greater computational storage and burden. The starting solution is often imposed near the lower limits, which again would increase computational burden and time and create rather complexity^31,32^. This complexity is mainly because the range between the upper and lower limits is often broad, aiming to ensure convergence. Additionally, the location of the optimal solution within the search space is usually ambiguous.

Despite the significance of initialization in the optimization process, it has received less attention in the existing literature. Previous studies have primarily focused on two aspects: first, employing optimization methods to fine-tune various hyperparameters of AI models to leverage their strengths and enhance prediction accuracy, and second, utilizing AI models to guide the setting of meta-heuristic parameters to improve search capability.

**Fig. 2:**
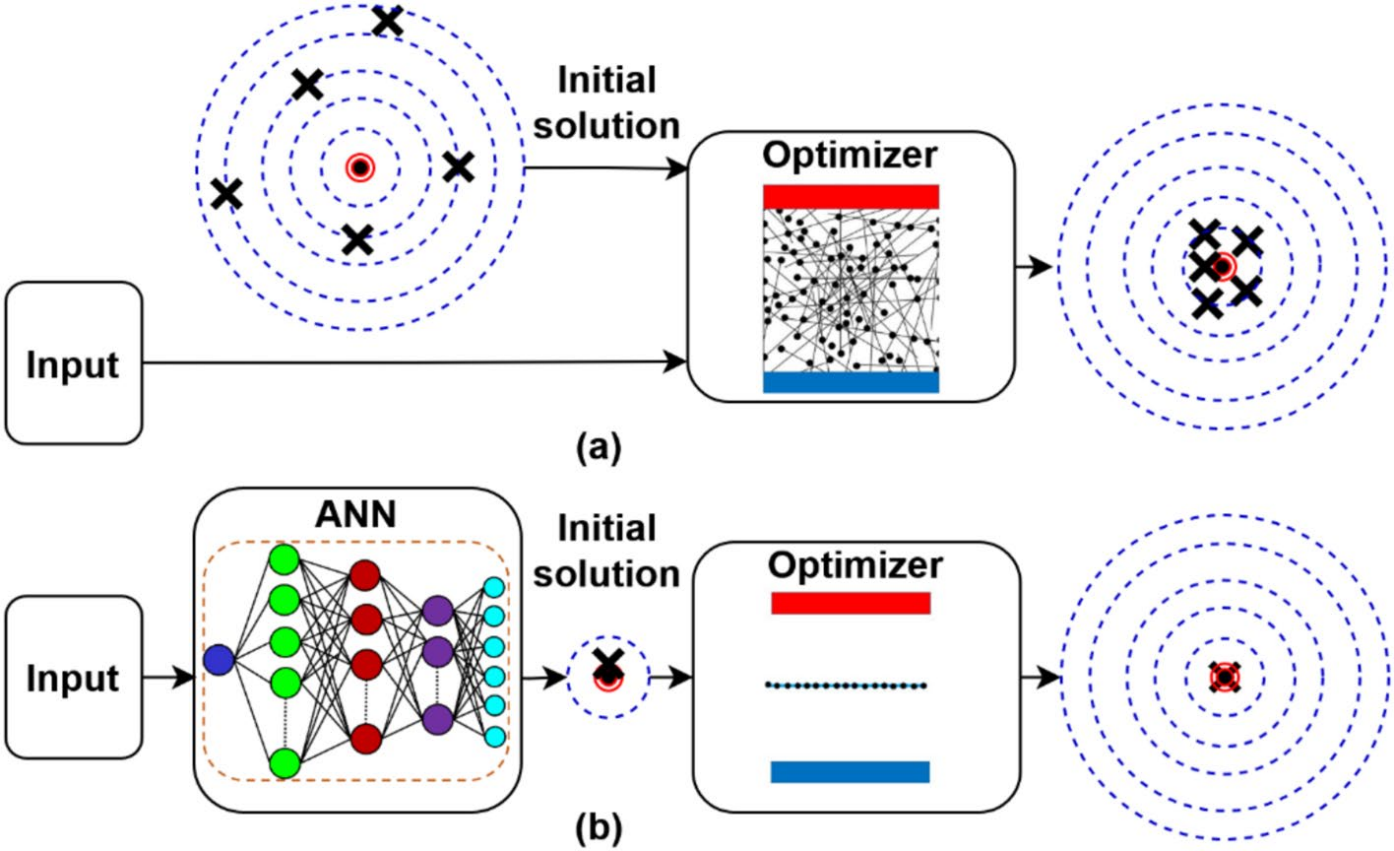
A comparative schematic of the proposed and traditional methods (**a**) Traditional optimizer and (**b**) AI-GPSed optimizer.

The key contributions of the proposed AI-GPSed optimizer are illustrated in Fig. 2, which are:

- Generating an elaborated initial solution driven by the problem/system under concern. This solution is derived from numerous AI training trials for the investigated problem/system, which boosts the possibility of global convergence and reduces computational time and burden.
- Determining the optimal range for the boundaries, which is typically around 80% narrower than traditional optimizers. These boundaries, again, accelerate convergence and minimize the execution time.

Figure 2 provides a comprehensive graphical comparison between a traditional optimizer and the proposed AI-GPSed optimizer.

The initial solution for the meta-heuristic optimizers, as shown in Fig. 2a, is generated randomly, irrespective of whether it is supplied by the user or internally generated. Moreover, as illustrated, there is no guarantee of convergence to the global optimal, which is the solution inside the red circle. Furthermore, the optimizer could be trapped in a local optimum. The AI-GPSed optimizer Fig. 2b generates the initial solution via its AI phase. This approach ensures global convergence under all operating scenarios. Figure 2 shows that the proposed technique relates the starting solution and the system/problem under concern, guaranteeing convergence to the global optimal.

**Fig. 3:**
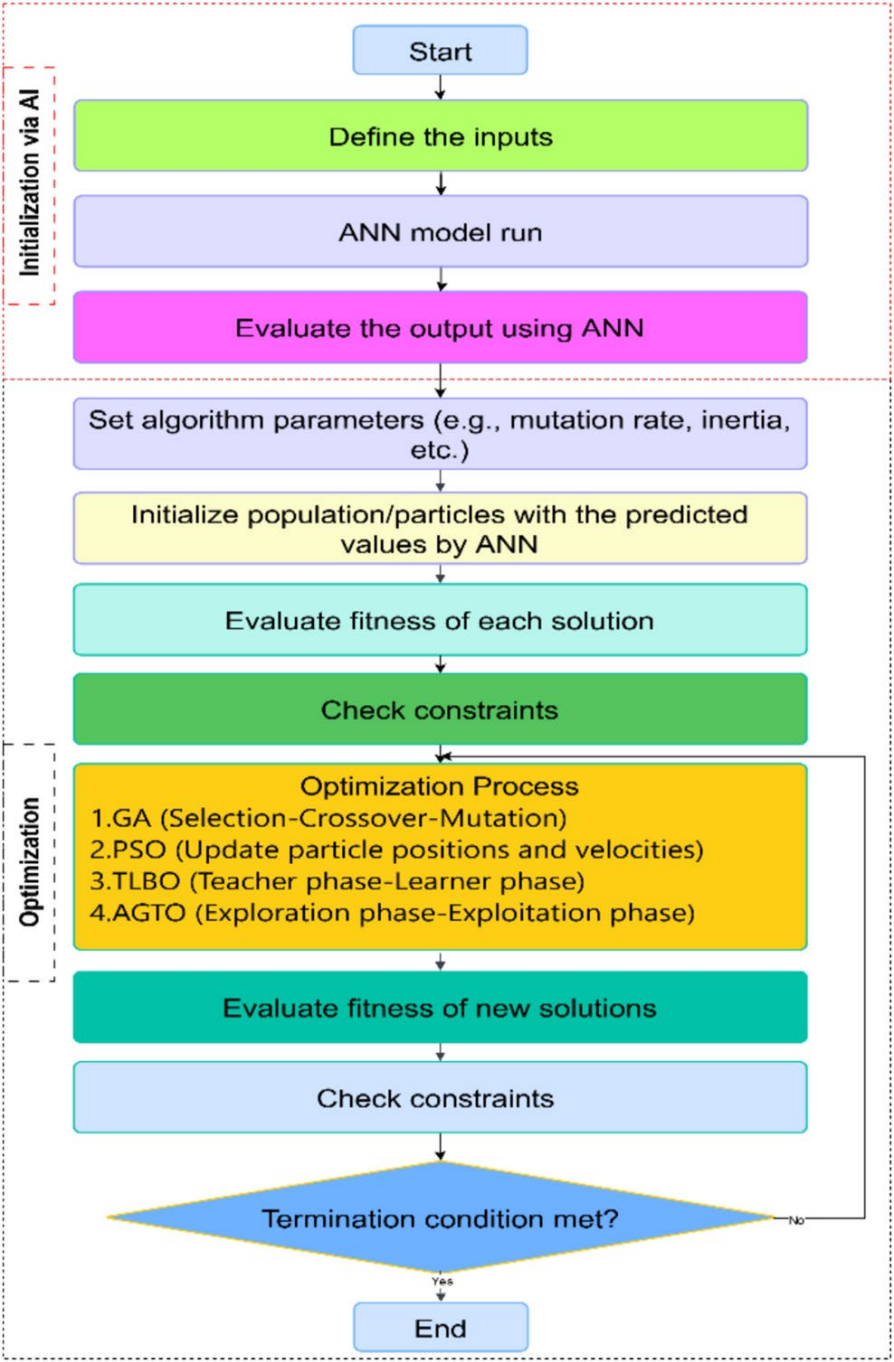
Flowchart of the ANN-various optimization techniques.

Figure 3 illustrates the flowchart of the proposed AI-GPSed optimizer, comprising two distinct phases. Moreover, the pseudocode for the AI-GPSed optimizer is depicted in Algorithm 1.

**Algorithm 1:**
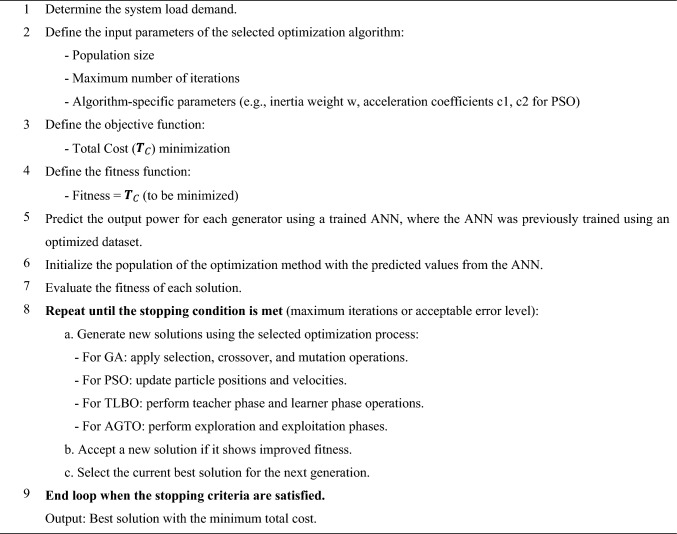
Pseudocode of AI-GPSed optimizer.

Figure 3 and Algorithm 1 show that unlike traditional optimization methods, the proposed approach does not begin randomly but starts from a solution predicted by the AI phase. This method also imposes narrower search boundaries, ensuring a more focused and accurate search near the optimal solution. It helps to reach the optimal solution faster. As a result, the solution tends to be more stable and cost-effective. Additionally, since multiple runs are not required, this targeted method is more efficient and well-suited for many applications.

The proposed AI-GPSed optimizer has two phases, as shown in Fig. 2b. These phases are themed: Phase 1 and Phase 2.

### Phase 1

AI, particularly through ML and ANN, serves as a guide for the optimization process by providing a starting point close to the global optimal solution. This guideness, in turn, narrows the search scope significantly. By leveraging effective prior training, AI restructures the optimization process, enabling faster searches and reducing the complexity of the search effort.

### Phase 2

This phase is carried out using any meta-heuristic optimizer, as the results will show. First, the parameters for the selected method are defined, and the process begins with an initial population or particles generated from the previous phase. The optimization method then proceeds through its standard steps: evaluating the quality of each solution, checking the imposed constraints, and updating the solutions based on the method’s specific techniques. Then, the quality of each solution is reassessed; the constraints are verified again. Finally, the stopping condition is tested, which involves reaching the optimal solution or completing the set number of iterations. If neither stopping condition is met, the steps are repeated until one is satisfied.

## Problem description

The ED problem is considered when evaluating AI-GPSed optimizers. The IEEE 30-bus system is used as the test case to investigate ED via the advised AI-GPSed optimizer. Since ED is fundamentally an optimization problem, the optimization process plays a crucial role in its solution. ED involves determining the optimal generation levels for a group of generators to meet specific load demand within the framework of minimizing the total cost of generation, emission cost, and power losses as an objective function while adhering to the imposed constraints, including generation limits^18^. From the above discussion, it could be concluded that the proposed ED problem provides a promising framework for evaluating the proposed method.

### Objective function

1 $${{\varvec{T}}}_{C}=\sum_{i=1}^{N} {a}_{i}{\left({P}_{i}\right)}^{2}+{b}_{i}{P}_{i}+{c}_{i }+{h}_{i}*\left({\sum }_{i=1}^{N} \left({d}_{i}{\left({P}_{i}\right)}^{2}+{e}_{i}{P}_{i}+{f}_{i}\right)\right)$$

2 $${h}_{i}=\frac{{F}_{c}\left({P}_{i}^{Max }\right)}{{E}_{T}\left({P}_{i}^{Max }\right)}=\frac{\left({a}_{i}{\left({P}_{i}^{Max }\right)}^{2}+{b}_{i}{P}_{i}^{Max }+{c}_{i}\right)}{\left({d}_{i}{\left({P}_{i}^{Max }\right)}^{2}+{e}_{i} {P}_{i}^{Max }+{f}_{i}\right)}$$

The total generation cost, $${T}_{C}$$(in $/h), is the sum of the fuel cost for each generating unit $$i$$ (in $/h) and the emission cost (in $/h). Here, $${P}_{i}$$​ represents the output power of unit $$i$$ (in MW), and N is the number of generating units. The fuel cost is determined by the coefficients, $${a}_{i}, {b}_{i},\text{ and }{c}_{i}$$, while the emission cost is influenced by the penalty factor $${h}_{i}$$​and the emission coefficients $${d}_{i}, {e}_{i},\text{ and }{f}_{i}$$​. Additionally*,* $${P}_{i}^{\text{Max }}$$​indicates the maximum power generation limit for unit $$i$$ ^11^.

### Constraints

*Power balance constraints*

3 $$\sum_{i=1}^{N} {P}_{i}-{P}_{L}-{P}_{D}=0$$

4 $${P}_{L}=\sum_{i=1}^{N} \sum_{j=1}^{N} {P}_{i}{B}_{ij}{P}_{j}+\sum_{i=1}^{N} {B}_{0i}{P}_{i}+{B}_{00}$$

where ${P}_{D}$ is the load demand (MW), ${P}_{L}$ is the power loss (MW), and ${B}_{ij},{B}_{oi}{B}_{oo}$ are the transmission loss coefficients ^11^.

*Generator operating limits constraints*

5 $${P}_{i}^{Min}\le {P}_{i}\le {P}_{i}^{Max };i=1,\dots ,N$$

where ${P}_{i}^{Min},{P}_{i}^{Max }$ are the lower and upper limits of generated power by unit $i$ ^11^.

### System under concern

**Fig. 4:**
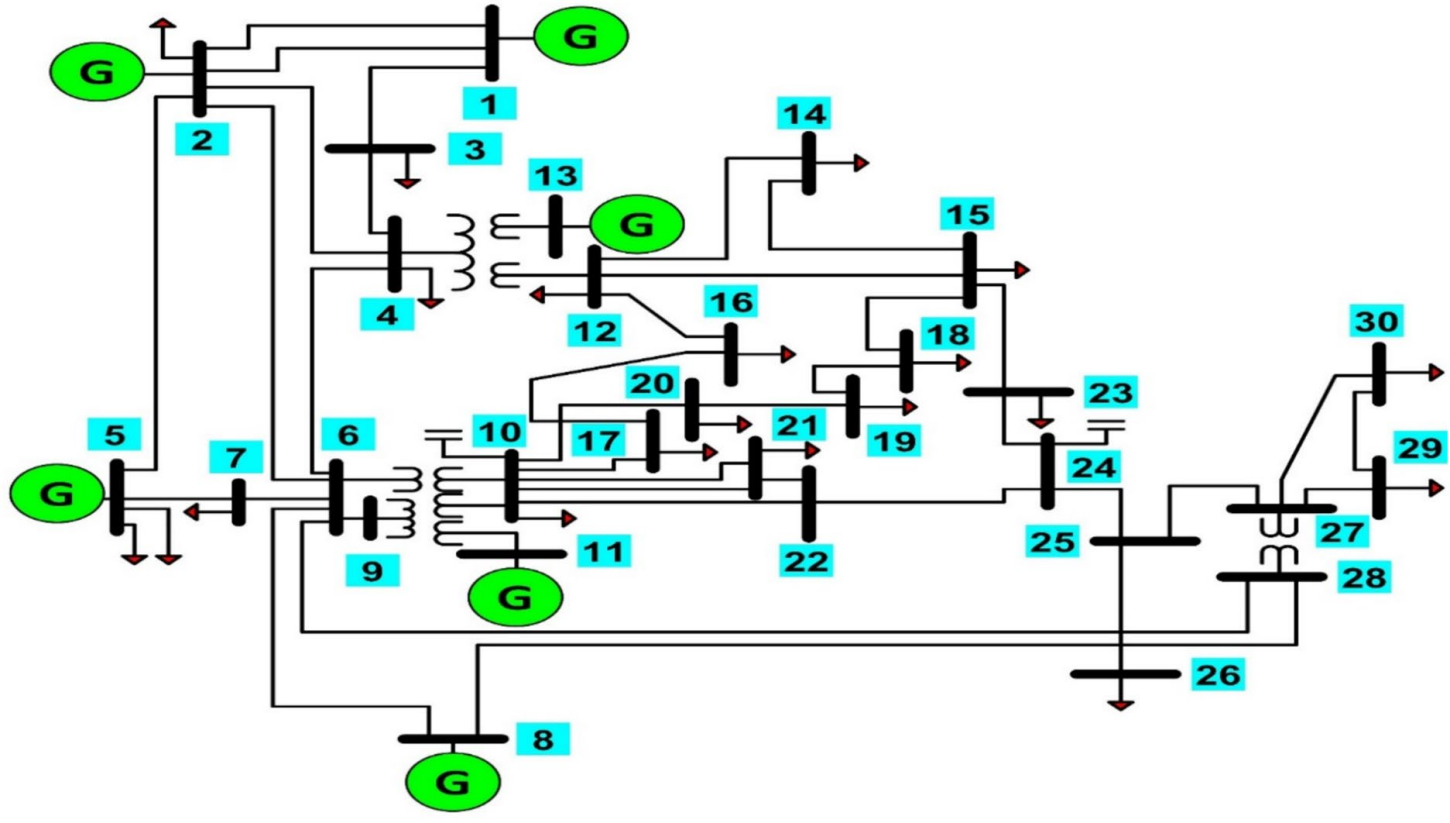
Single line diagram of IEEE standard 30-bus system.

The IEEE 30-bus system is a widely used benchmark for evaluating the efficiency of power systems and transmission networks. As shown in Fig. 4. The system consists of 30 buses, including six generators with a combined generation capacity of 1350 MW. Detailed parameters for the six generating units are presented in Table 1, while the system’s loss coefficients can be found in Table 2^10,11^.

**Table 1:** Generating unit test data of the IEEE 30‐bus system.

Gen. Unit	*a*_*i*_ ($/MW^2^.h)	*b*_*i*_ ($/MW.h)	*c*_*i*_ ($/h)	*d*_*i*_ ($/MW^2^.h)	*e*_*i*_ ($/MW.h)	*f*_*i*_ ($/h)	*P*_*i*_^*Min*^ (MW)	*P*_*i*_^*Max*^ (MW)
1	0.1525	38.54	756.8	0.0042	0.33	13.86	10	125
2	0.106	46.16	451.32	0.004	0.33	13.86	10	150
3	0.0208	40.159	1049.99	0.00683	− 0.5455	40.27	35	225
4	0.0355	38.31	1234.5	0.0068	− 0.5455	40.27	35	210
5	0.0211	36.328	1658.6	0.0046	− 0.5112	42.7	130	325
6	0.0179	38.27	1356.7	0.0042	− 0.5112	42.7	125	315

**Table 2:** Parameters of transmission loss for IEEE 30 bus 6-unit.

B-loss coefficient					
0.14E-04	0.17E-04	0.15E-04	0.19E-04	0.26E-04	0.22E-04
0.17E-04	0.60E-04	0.13E-04	0.16E-04	0.15E-04	0.20E-04
0.15E-04	0.13E-04	0.65E-04	0.17E-04	0.24E-04	0.19E-04
0.19E-04	0.16E-04	0.17E-04	0.71E-04	0.30E-04	0.25E-04
0.26E-04	0.15E-04	0.24E-04	0.30E-04	0.69E-04	0.32E-04
0.22E-04	0.20E-04	0.19E-04	0.25E-04	0.32E-04	0.85E-04

### Study cases

In this research, the proposed AI-GPSed optimizer combines ANN with one of the four distinctive optimization algorithms (GA, PSO, TLBO, and AGTO) each time. AI-GPSed optimizer aims to create a reliable and effective ED technique capable of achieving the lowest generation cost while maintaining stable performance.

The proposed method begins by constructing a neural network with four layers (97-81-76-6). The network has the required load as input and produces six values as output, representing the power generated by each generator. It is trained using data from prior optimization runs. These data result from PSO on the same system, which considers identical fuel costs and emissions. The PSO method is utilized for its high efficiency and rapid convergence, significantly reducing computation time. Meanwhile, the role of ANNs is to guide the optimizer toward the optimal solution by bounding the search space to a narrower region near the expected optimum rather than initiating the search randomly in a wide range. Any of the four optimization algorithms can be used to generate training data, as all can yield reasonable solutions.

A total of 2012 data sets were collected to capture the relationship between the input and optimized output. This dataset was randomly split into 60% for training, 20% for validation, and 20% for testing. It has utilized distinct datasets for different experiments and ensured no overlapping data was used across multiple tests. The training used backpropagation to minimize the error between predicted and target values enhancing accuracy. The sigmoid activation function was applied to all hidden layers due to its computational efficiency and ability to alleviate the vanishing gradient problem. A linear activation function was used in the output layer as the target variable (generation power for six generators).

The model was trained using the Adam optimizer over 1500 epochs. The Mean Squared Error (MSE) was adopted as the loss function, which is well-suited for regression tasks. A learning rate of 0.01 was selected to ensure stable convergence without oscillations or stagnation during training. Hyperparameter tuning was initially performed manually, followed by using GridSearchCV from the sklearn library in combination with KerasClassifier to optimize the model parameters. For performance evaluation and error analysis, Mean Squared Error (MSE), Root Mean Squared Error (RMSE), and Mean Absolute Error (MAE) were used.

This study uses the same ANN for all optimization methods within the same narrow search framework. The basic driver for using the same ANN for the four optimizers under concern for the same operating scenarios is to achieve the principle of equivalence for a fair comparison. Therefore, two ANNs were used in this study: one for a lossless study and the other for a lossy study due to the difference in the maximum load that can be fed. Each network is applied for GA, PSO, TLBO, and AGTO without modification and/or alteration. Upon completion of the ANN construction, it is integrated with the optimization method to guide the search within a more focused range. This integration enhances the ability to identify optimal solutions and improves the overall efficiency of the system.

The effectiveness of the proposed approaches was tested using four different optimization methods, implemented in MATLAB, under load demand of 700 MW and 1000 MW across 4 test cases. Each test case included two scenarios: one for a 700 MW load and the other for a 1000 MW load. Within each scenario, two distinct operating conditions were analyzed. The first condition considered operation without losses, while the second accounted for operation with losses. Consequently, the results for each scenario will include the two operating conditions:

- Test case 1: ANN-GA method.
- Test case 2: ANN-PSO approach.
- Test case 3: ANN-TLBO technique.
- Test case 4: ANN-AGTO method.

## Results and discussions

### Test case 1: the performance of the ANN-GA method

#### Scenario I-1: the load is 700 MW, with and without losses

This test assesses the performance of the proposed ANN-GA and the traditional GA to determine the most efficient and reliable technique for minimizing the total generation cost and emissions from fuel combustion. Two scenarios were analyzed: one considering transmission losses and the other without.

**Fig. 5:**
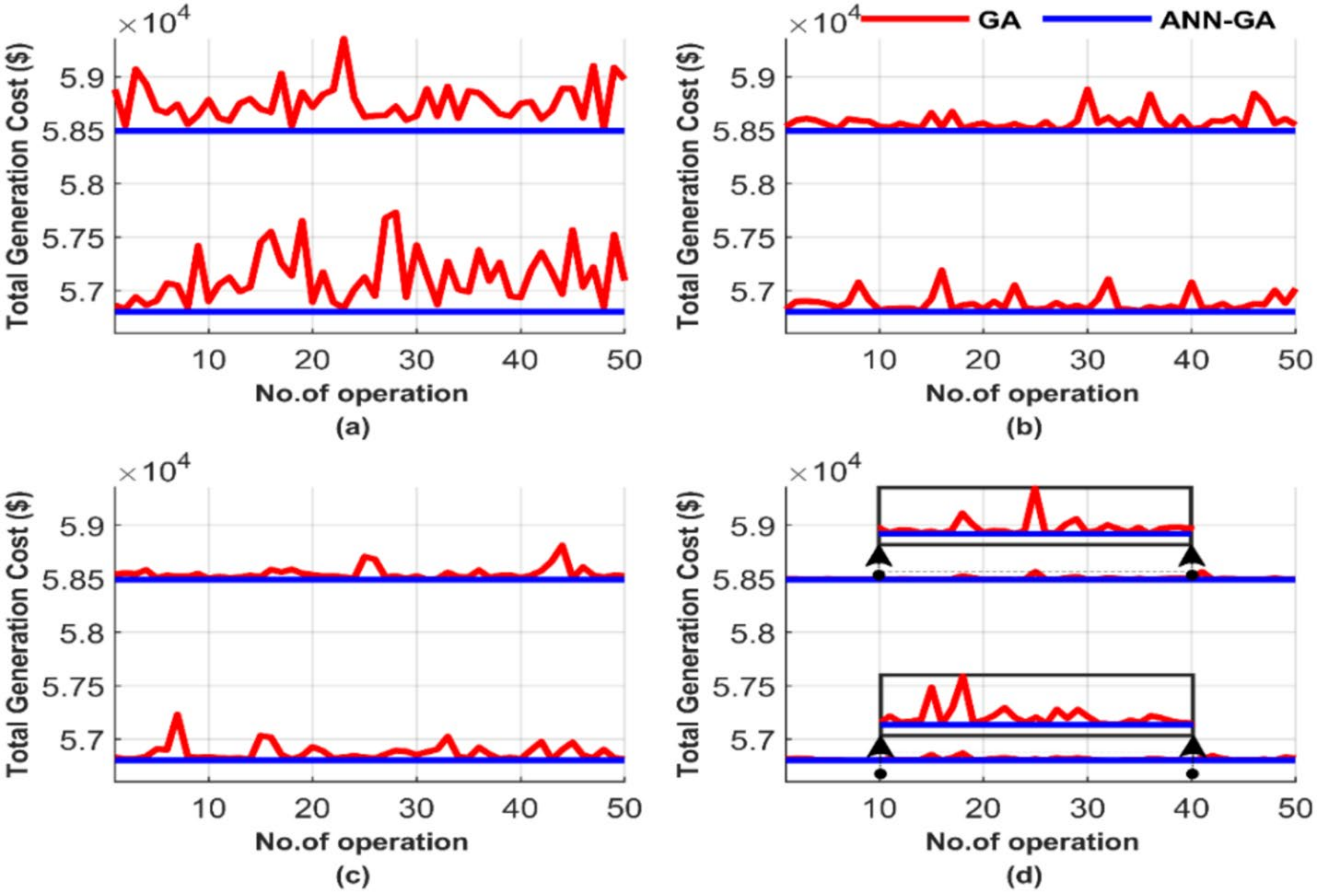
Comparison of cost curves over 50 runs: (**a**) 100 iterations, 25 populations, (**b**) 100 iterations, 50 populations, (**c**) 1000 iterations, 50 populations, (**d**) 1000 iterations, 100 populations.

Figure 5 compares the performance between the GA and the proposed ANN-GA over 50 runs, highlighting differences in the number of iterations and population size. Figure 5 comprises four subfigures: (a), (b), (c), and (d), each subfigure representing a different combination of iterations and population sizes for the 700 MW load. This setup tests the sensitivity of both GA and the proposed ANN-GA over 50 runs. The program is executed 50 times to examine the constancy.

Figure 5 shows that the proposed ANN-GA always convergences to the same solution, the global optimum, irrespective of the number of iterations or population. This figure indicates the effectiveness of the proposed ANN-GA. Figure 5 shows that the quality of the GA solution improves as the number of iterations and population increase. It is evident by comparing the graph in Fig. 5d and the other graphs in the same figure. GA is highly sensitive to changes in population size and the number of iterations. Meanwhile, the proposed ANN-GA produces a constant solution, the global optimum, with the least computation requirements. Moreover, the quality of the solution from the proposed ANN-GA is much better than that of GA, Fig. 5. This improved performance of the proposed AI-GA is attributed to ANN, which serves as a guide to the quickest and most stable path to the solution for GA, unlike the traditional approach that always starts with random values.

**Fig. 6:**
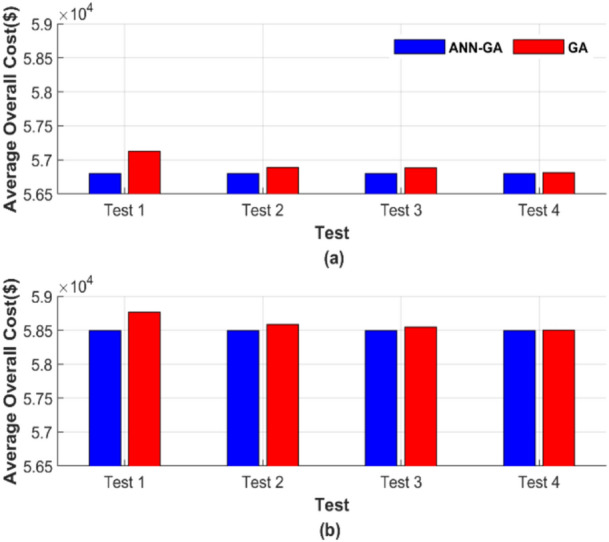
Average overall cost for ED problem: (**a**) without losses and (**b**) with losses.

Figure 5 presents the total generation cost for each of the 50 runs under different operating conditions. The four tests depicted in Fig. 5 correspond to four distinct operating conditions, with the results of each test shown individually in a separate graph. The average of the total generation cost for each test was calculated to facilitate the comparison between these tests, and the results are displayed in Fig. 6, which consists of two subfigures: one showing the average total generation cost for the four operating conditions without losses, and the other showing the results with losses.

Figure 6 confirms the observations discussed earlier in Fig. 5. As explained, the ANN-GA method consistently provides a lower and nearly constant cost across all operating cases. In Fig. 6a, the cost is 56,801.16 $, while in Fig. 6b, it is 58,491.42 $. In contrast, the cost of the GA method varies depending on the operating conditions. Even under the best-case scenario, GA barely approaches the performance of the target method, yielding a cost of 56,812.22 $, 58,500.99 $ in both Fig. 6a and b, which is notably higher than the cost achieved by the ANN-GA method. The graphical representation of the average total costs in Fig. 6 further confirms the constancy and efficiency of ANN-GA, in contrast to GA, which remains sensitive to population size and the number of iterations. The proposed AI-GA offers less cost than GA despite the increase in population size and the number of iterations.

**Fig. 7:**
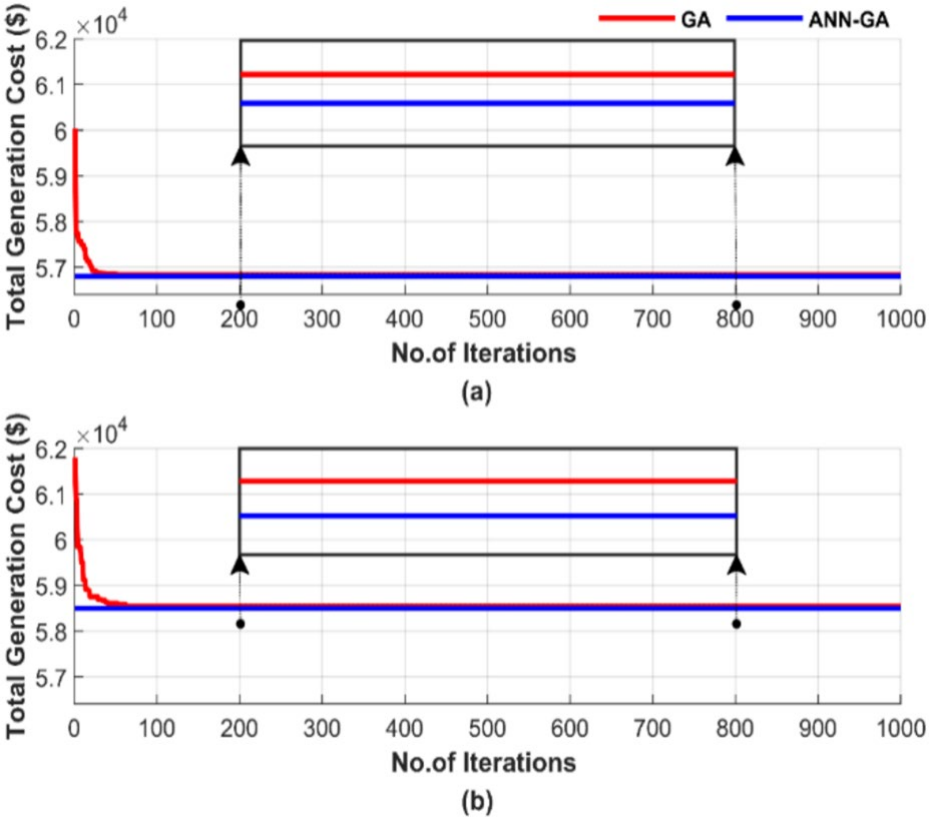
Cost convergence of two algorithms for ED problem over a single run with 1000 iterations and 50 populations: (**a**) without losses and (**b**) with losses.

To evaluate the convergence rate, Fig. 7 shows the random behavior of the algorithms in terms of total generation cost during a single run, using a population size of 50 and 1000 iterations. A population size of 50 was selected as a compromise between the 25 and 100 population sizes. The number of iterations for this run is set to 1000, a large value chosen to ensure the convergence of both techniques. Figure 7 presents two subfigures: one showing the total generation cost without losses and the other showing the results with losses.

Figure 7 clearly illustrates the difference in performance. The proposed ANN-GA achieves a 0.14% lower cost than GA in Fig. 7a and a 0.1% lower cost than GA in Fig. 7b. ANN-GA requires 100% fewer iterations compared to GA in both cases. This results demonstrates the ANN-GA’s success in achieving lower costs in significantly less time.

**Fig. 8:**
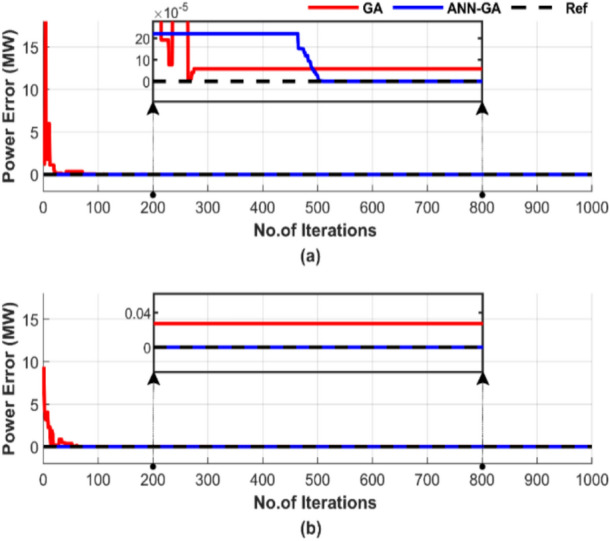
Power mismatch convergence of two algorithms for ED problem over a single run with 1000 iterations and 50 populations: (**a**) without losses and (**b**) with losses.

The effectiveness of ED is assessed by examining the power balance between demand and generation under two conditions: with and without losses. In the case of ED with transmission line losses, the power balance is defined as the equilibrium between generation and the sum of load demand and losses. The power mismatches for both operating conditions are shown in Fig. 8 for a randomly selected single run with a population size of 50 and 1000 iterations. The figure is intended to verify whether the power balance is achieved with a power mismatch of zero and determine how many iterations are required to reach that point.

Figure 8 demonstrates that ANN-GA achieves a better power balance by producing output that closely matches the load, a power mismatch of zero. In Fig. 8a, ANN-GA outperforms GA by stabilizing more quickly. ANN-GA stabilizes after 500 iterations; this time is spent searching for a solution closer to ANN’s. Additionally, Fig. 8b highlights the faster response of ANN-GA in achieving approximately perfect power balance, surpassing GA in performance.

#### Scenario II-1: the load is 1000 MW, with and without losses

This test assesses the performance of the ANN-GA and GA methods for a load of 1000 MW. It evaluates the efficiency and effectiveness of the proposed method at a different load level. The test includes two cases: with and without transmission losses. It was conducted at four distinct times, each with different parameters for the number of iterations and population size.

**Fig. 9:**
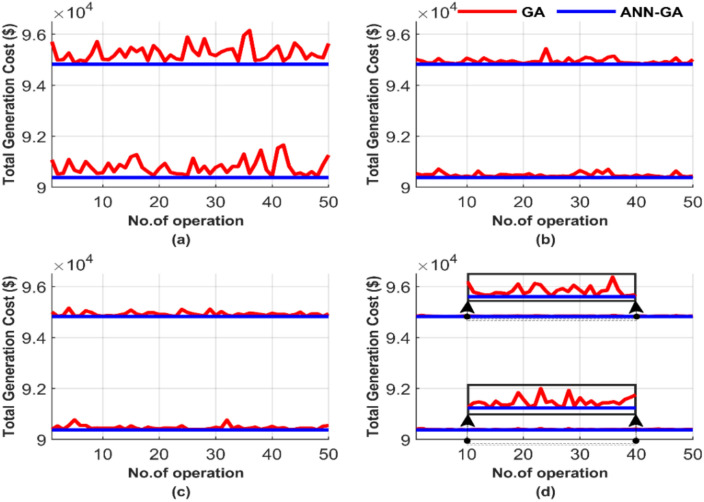
Comparison of cost curves over 50 runs: (**a**) 100 iterations, 25 populations, (**b**) 100 iterations, 50 populations, (**c**) 1000 iterations, 50 populations, (**d**) 1000 iterations, 100 populations.

Figure 9 graphically compares the performance of the GA and the proposed ANN-GA over these 50 runs, highlighting the impact of different iterations and population sizes. The results are illustrated in Fig. 9: (a) for 100 iterations and 25 populations, (b) for 100 iterations and 50 populations, (c) for 1000 iterations and 50 populations, and finally (d) for 1000 iterations and 100 populations. Each case was run 50 times, accounting for population size and iteration number variations.

In Fig. 9, the sensitivity of GA to population size and the number of iterations is evident as its performance fluctuates. Additionally, the performance of GA improves with an increase in population size and the number of iterations. However, this comes at the cost of increased computation time and resource requirements. Even with a large population size and many iterations, the solution of GA remains less viable compared to the proposed AI-GA. In contrast, the proposed ANN-GA delivers near-constant results regardless of population size and the number of iterations. This consistency is attributed to its guided approach, which leads to faster and more reliable solutions, unlike the traditional GA method that starts with random values.

**Fig. 10:**
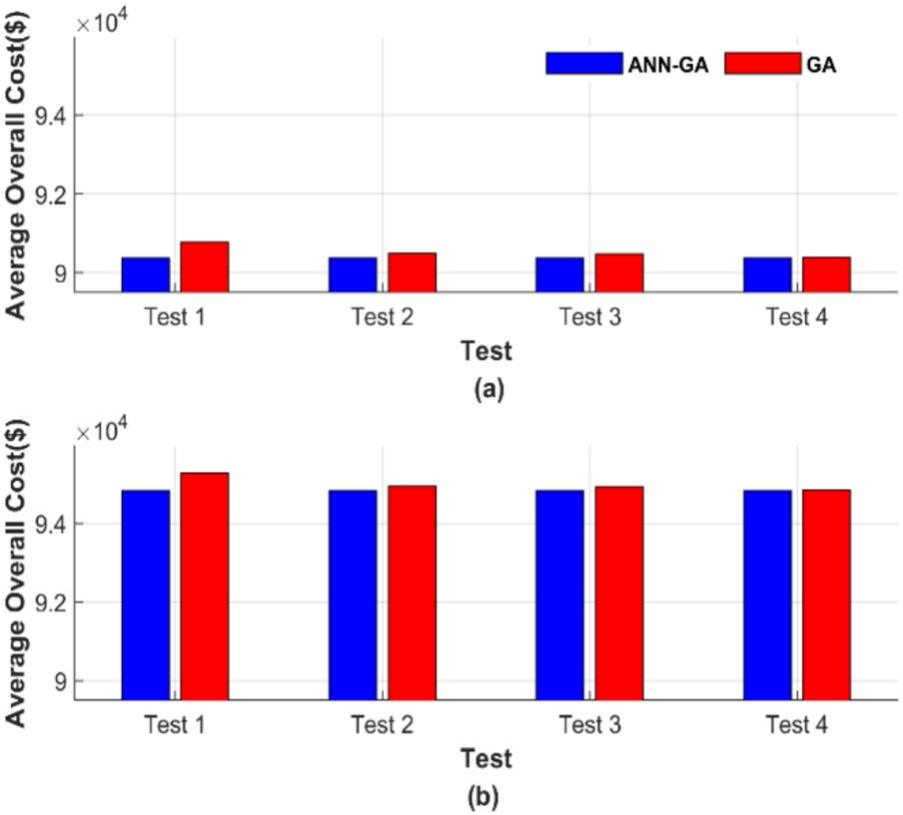
Average overall cost for ED problem: (**a**) without losses and (**b**) with losses.

Figure 10 provides a graphical representation of the average cost for each of the 50 runs under different operating conditions. Figure 10 contains two subfigures: Fig. 10a displays the average total generation cost for the four operating conditions without losses, while Fig. 10b presents the results with losses.

Figure 10 shows that the ANN-GA method outperforms in achieving a lower generation cost across all operating conditions and scenarios. ANN-GA method maintains almost equal costs in all operating conditions, with values of 90,378.06 $ and 94,826.30 $ in Fig. 10a and b. In contrast, under its best operating parameters, GA achieves costs of 90,392.24 $ and 94,838.66 $, which are higher than the ANN-GA method. This further validates the constancy of the ANN-GA in achieving the lowest cost while highlighting the sensitivity of the GA to changes in population size and iterations.

**Fig. 11:**
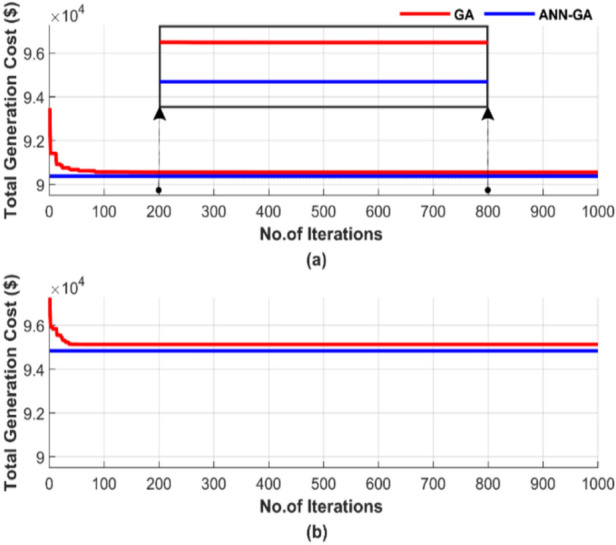
Cost convergence of two algorithms for ED problem over a single run with 1000 iterations and 50 populations: (**a**) without losses and (**b**) with losses.

Figure 11 depicts the convergence rate of the algorithms based on a randomly selected single run, using a population size of 50 and 1000 iterations. The results are presented in two subfigures: Fig. 11a presents the total generation cost without losses, and Fig. 11b displays the results with losses.

Figure 11 also highlights the ability of ANN-GA to reduce costs more effectively, responding faster and more consistently than GA. ANN-GA method achieves a 0.1% cost reduction compared to GA in Fig. 11a and a 0.11% reduction compared to GA in Fig. 11b, while ANN-GA completes the optimization with 100% fewer iterations than GA in both cases.

**Fig. 12:**
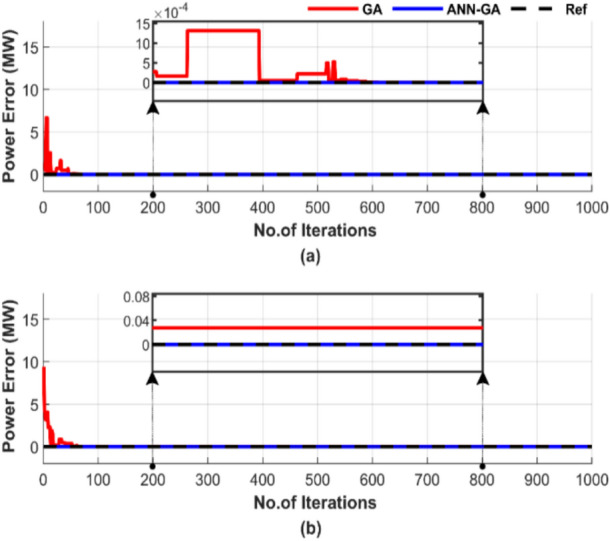
Power mismatch convergence of two algorithms for ED problem over a single run with 1000 iterations and 50 populations: (**a**) without losses and (**b**) with losses.

Figure 12 shows the power mismatches between the generated power and load demand for a randomly selected single run, using a population size of 50 and 1000 iterations, with and without losses.

Figure 12 shows that both methods achieve power balance in Fig. 12a, but ANN-GA is completely stable from the start, while GA takes 600 iterations to achieve that. Additionally, Fig. 12b highlights the inability of GA to reach the optimum power, unlike ANN-GA.

Figures 5, 6, 7, 8, 9, 10, 11 and 12 indicate that the proposed AI-GA is more efficient and reliable than GA. Moreover, the proposed optimizer converges much faster than GA.

### Test case 2: the performance of the ANN-PSO method

#### Scenario I-2: the load is 700 MW, with and without losses

The objective of this test is to evaluate ANN-PSO and the traditional PSO. Two scenarios were conducted: one considering losses and the other without. This assessment was carried out across four different operating conditions, each with varying numbers of iterations and particles, to meet a load demand of 700 MW. Results were obtained by running each case 50 times, considering the changes in particle number and iterations.

**Fig. 13:**
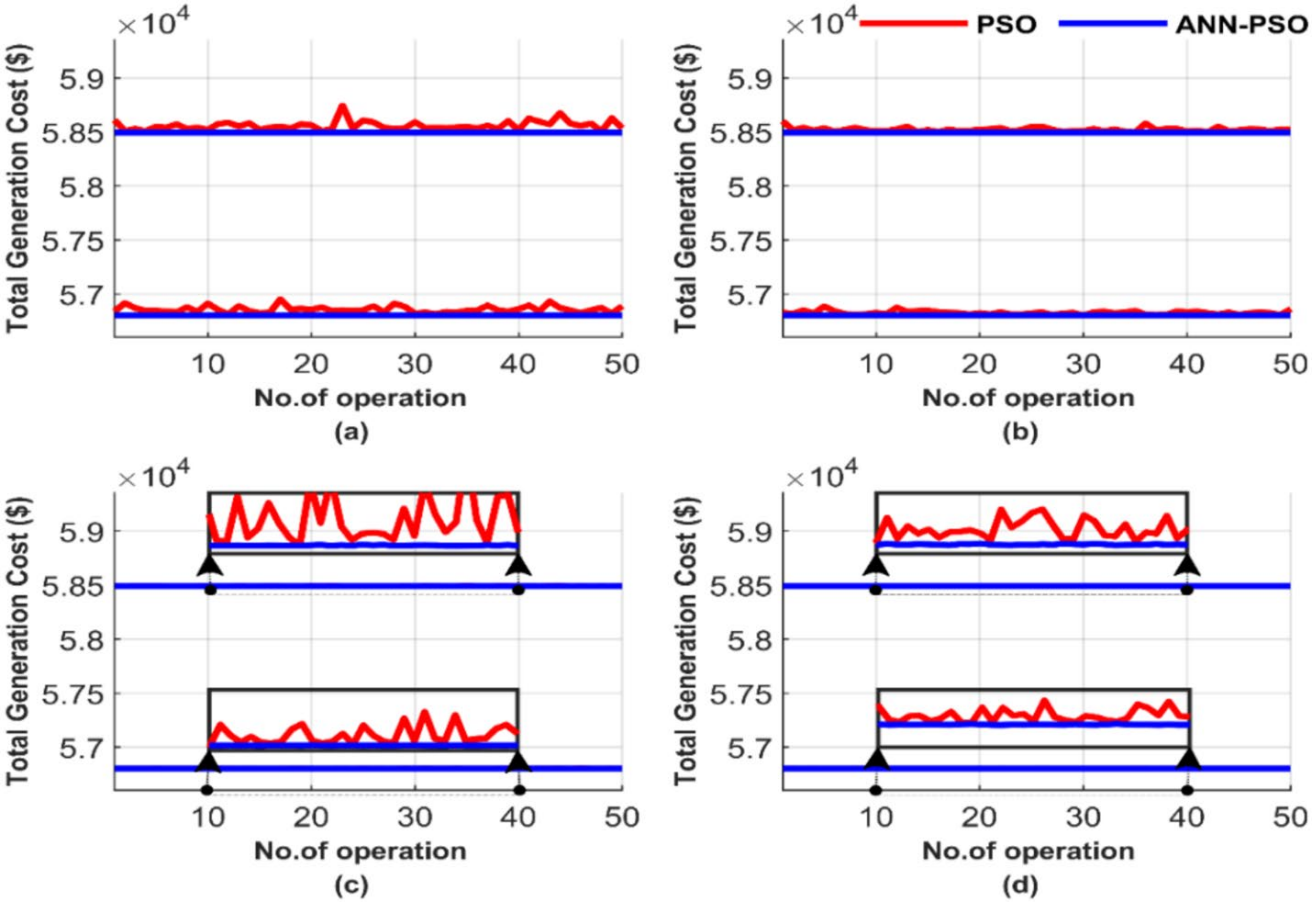
Comparison of cost curves over 50 runs: (**a**) 100 iterations, 25 particles, (**b**) 100 iterations, 50 particles, (**c**) 1000 iterations, 50 particles, (**d**) 1000 iterations, 100 particles.

Figure 13 compares the performance of the PSO and the proposed ANN-PSO across these 50 runs, emphasizing differences in iterations and particle numbers. The outcomes are displayed in Fig. 13, showing: (a) for 100 iterations and 25 particles, (b) for 100 iterations and 50 particles, (c) for 1000 iterations and 50 particles, and finally (d) for 1000 iterations and 100 particles.

The graphical representation of total costs over 50 runs for four different operating conditions in Fig. 13 demonstrates the strength and constancy of the PSO method. It shows that PSO is less sensitive compared to the GA method, which results from comparing Fig. 5 and Fig. 13. However, the proposed ANN-PSO produces the lowest cost irrespective of the population and/or the iteration number. This performance further confirms the constancy and efficiency of ANN-PSO in contrast to PSO. PSO is still sensitive to changes in the number of particles and iterations, which is evident from comparing the subfigures (a), (b), (c), and (d).

**Fig. 14:**
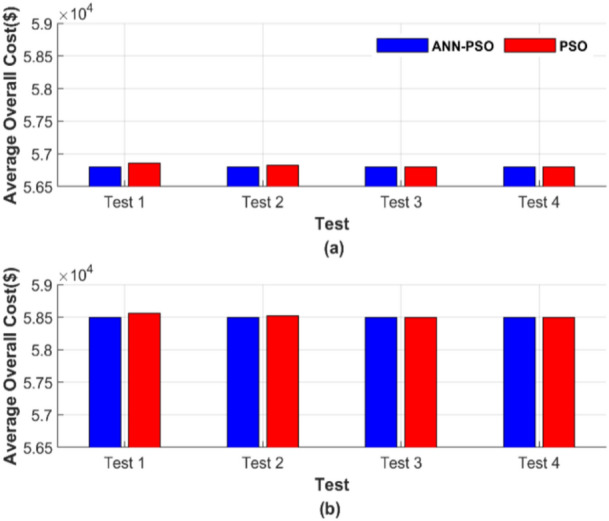
Average overall cost for ED problem: (**a**) without losses and (**b**) with losses.

Figure 14 presents a graphical display of the average total cost for each of the 50 runs. Figure 14a shows the average total generation cost for the four operating conditions without losses, while Fig. 14b displays the results with losses.

Figure 14 confirms the strength and robustness of the AI-PSO compared to a powerful optimization technique, such as PSO. However, the total cost of the PSO method is close to that of the ANN-PSO. The proposed ANN-PSO outperforms in achieving a lower cost with complete constancy across all operating conditions. The costs were 56,801.15 $ and 58,491.42 $ for the two operating scenarios, while the best operating costs for the PSO method were 56,801.27 $ and 58,491.57 $.

**Fig. 15:**
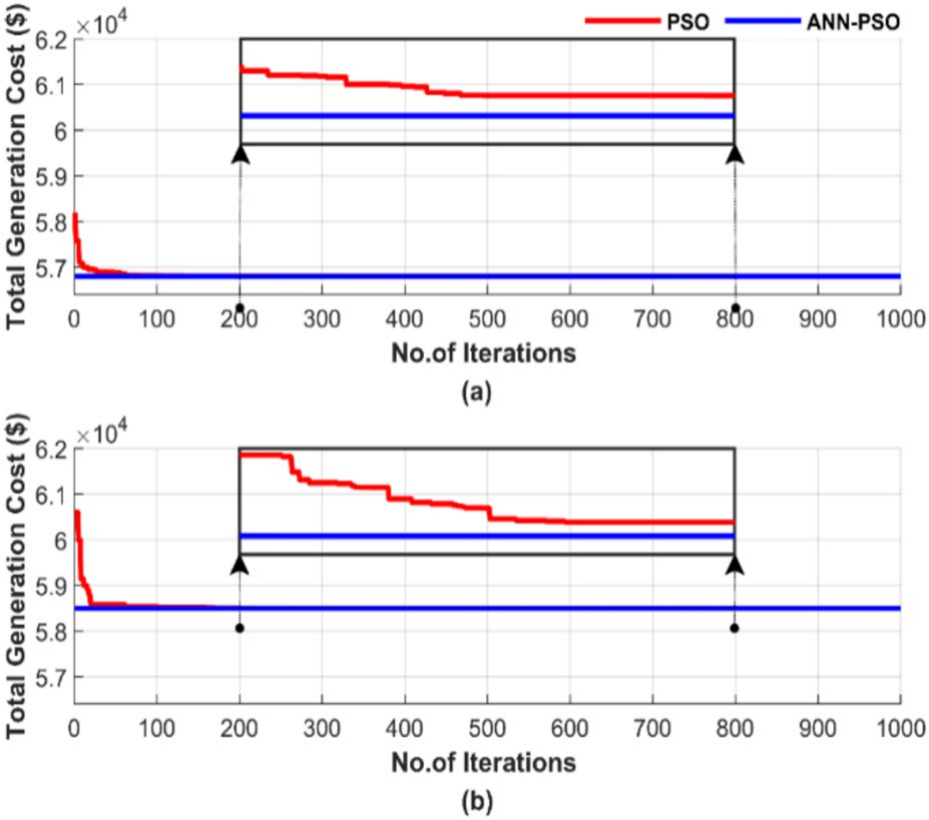
Cost convergence of two algorithms for ED problem over a single run with 1000 iterations and 50 particles: (**a**) without losses and (**b**) with losses.

Figure 15 illustrates the convergence of the algorithms based on a single randomly selected run, using a particle number of 50 and 1000 iterations. The results are depicted in two subfigures: Fig. 15a shows the total generation cost excluding losses, while Fig. 15b presents the cost including losses.

Figure 15 highlights the faster and more consistent cost reduction ANN-PSO achieves compared to traditional PSO. ANN-PSO method achieves a 0.001% cost reduction in both Fig. 15a and b. PSO required over 500 iterations to reach a stable value; the resulting cost was still higher than the proposed method, which appeared completely stable from the outset. Consequently, the ANN-PSO method achieved a lower cost in significantly less iterations.

**Fig. 16:**
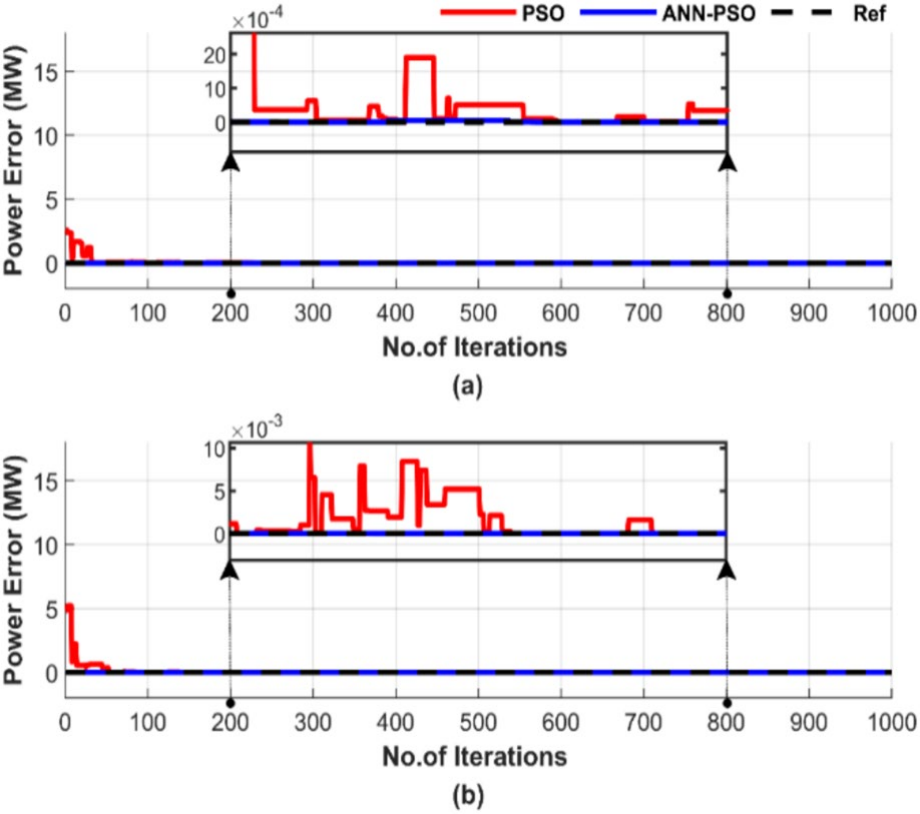
Power mismatch convergence of two algorithms for ED problem over a single run with 1000 iterations and 50 particles: (**a**) without losses and (**b**) with losses.

Figure 16 shows the power mismatches between the generated power and load demand during a single run with a particle number of 50 and 1000 iterations. Figure 16a displays the power mismatches for the test without losses, while Fig. 16b shows the mismatches for the test with losses.

Figure 16 shows that although both methods manage to balance power output, ANN-PSO reaches constancy much sooner than the PSO method, which suffers from difficulties in achieving this, even if the number of iterations reaches 700.

#### Scenario II-2: the load is 1000 MW, with and without losses

ANN-PSO had to be tested again at a load level of 1000 MW to confirm its quality and effectiveness. The evaluation was conducted across four operating conditions with varying numbers of iterations and particle sizes. Each case was run 50 times, accounting for particle differences and iteration numbers. Two scenarios were conducted: one considering losses and the other neglecting them.

**Fig. 17:**
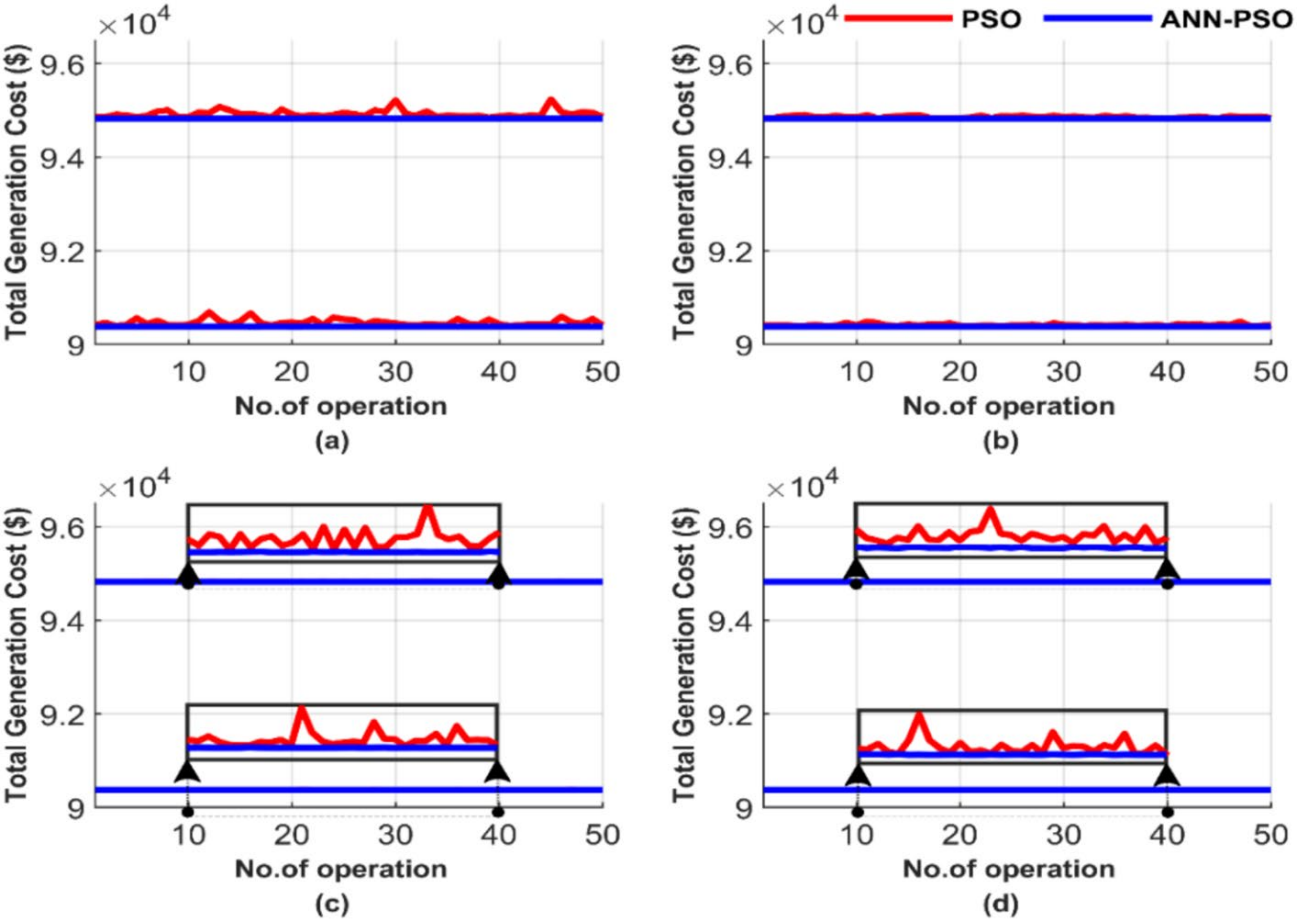
Comparison of cost curves over 50 runs: (**a**) 100 iterations, 25 particles, (**b**) 100 iterations, 50 particles, (**c**) 1000 iterations, 50 particles, (**d**) 1000 iterations, 100 particles.

Figure 17 compares the performance of both methods over these 50 runs, highlighting the impact of iteration and particle number variations. As illustrated in Fig. 17, the results include: (a) for 100 iterations and 25 particles, (b) for 100 iterations and 50 particles, (c) for 1000 iterations and 50 particles, and finally (d) for 1000 iterations and 100 particles.

The results still confirm the relative efficiency of the PSO method. However, the proposed ANN-PSO produces a much better performance than PSO. In Fig. 17, the ability of ANN-PSO to achieve the lowest cost with complete constancy is shown irrespective of particles and/or iteration number. In contrast, the performance of the PSO method depends on changes in particles and the number of iterations. The quality of the solution of PSO as GA improves with the increase in the number of particles/population and the number of iterations.

**Fig. 18:**
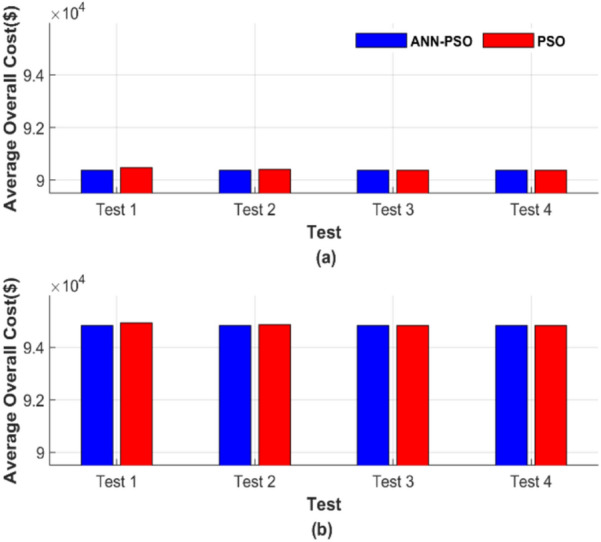
Average overall cost for ED problem: (**a**) without losses and (**b**) with losses.

Figure 18 illustrates the average total cost across 50 runs in a graphical format. Figure 18a represents the average total generation cost under four operating conditions without considering losses, while Fig. 18b shows the corresponding results with losses included.

Figure 18 demonstrates that the ANN-PSO method outperforms the PSO method by achieving lower costs with complete constancy across all operating conditions. The costs using the ANN-PSO method were 90,378.06 $ and 94,826.30 $ for the two operating scenarios, whereas the best operating costs achieved by the PSO method were 90,378.28 $ and 94,826.54 $, respectively.

**Fig. 19:**
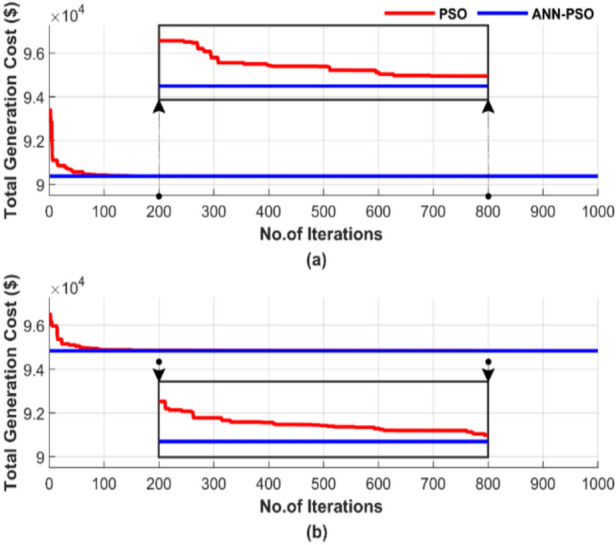
Cost convergence of two algorithms for ED problem over a single run with 1000 iterations and 50 particles: (**a**) without losses and (**b**) with losses.

Figure 19 depicts the random behavior of the ANN-PSO and PSO methods during a single run, using 50 particles and 1000 iterations. Figure 19a displays the total generation cost without considering losses, while Fig. 19b shows the cost, including losses.

Figure 19 confirms the superiority of the proposed ANN-PSO over the PSO algorithm, where the ANN-PSO achieved a 0.001% cost reduction compared to PSO in both Fig. 19a and b. Figure 19 also demonstrates ANN-PSO’s ability to achieve lower costs more quickly and with complete constancy. In contrast, the PSO algorithm requires over 600 iterations to stabilize.

**Fig. 20:**
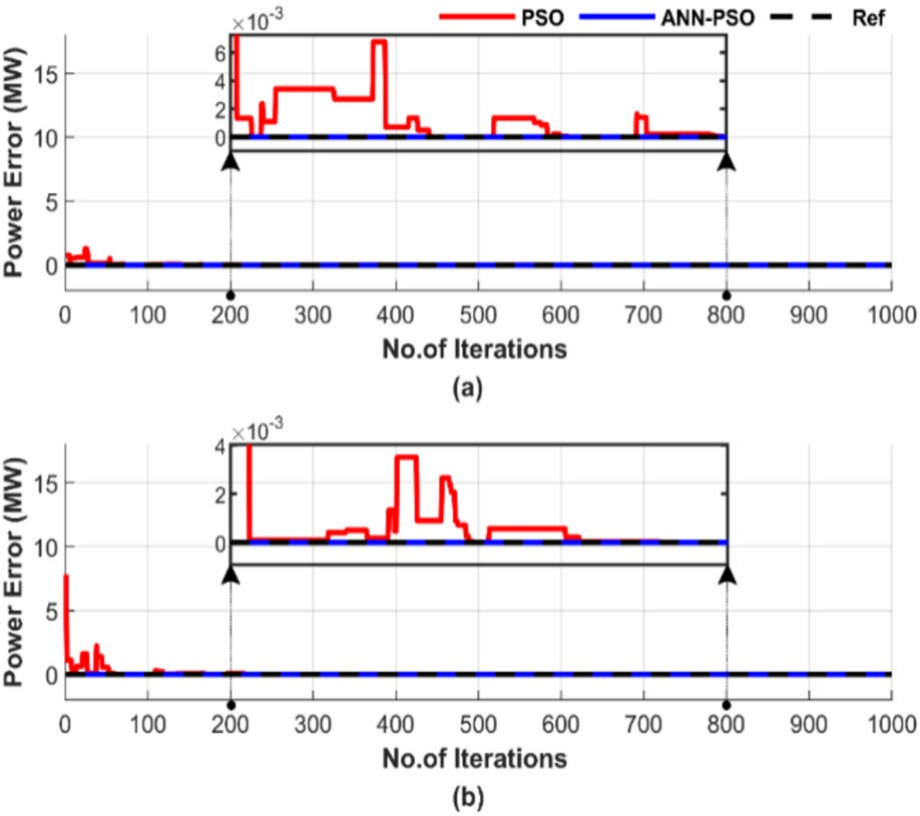
Power mismatch convergence of two algorithms for ED problem over a single run with 1000 iterations and 50 particles: (**a**) without losses and (**b**) with losses.

Figure 20 demonstrates the difference between the generated power and the load demand during a single run, using a particle number of 50 and 1000 iterations. Figure 20a shows the power mismatches excluding losses, while Fig. 20b represents them with losses included.

Figure 20 shows that ANN-PSO demonstrates complete constancy in achieving power balance, whereas the PSO method was late arriving and is characterized by complete inconstancy.

Figures 13, 14, 15, 16, 17, 18, 19 and 20 demonstrate that the proposed AI-PSO algorithm outperforms the standard PSO’s efficiency and reliability. Additionally, the proposed optimizer achieves significantly faster convergence compared to PSO.

### Test case 3: the performance of the ANN-TLBO method

#### Scenario I-3: the load is 700 MW, with and without losses

In this test, the proposed technique was applied to TLBO to evaluate the potential improvements that could occur and to extract the most stable and least cost method. The test was conducted across four scenarios with varying iterations and population sizes to achieve a 700 MW load demand, analyzing both with and without losses. Each scenario was executed 50 times, considering different population sizes and iteration counts.

**Fig. 21:**
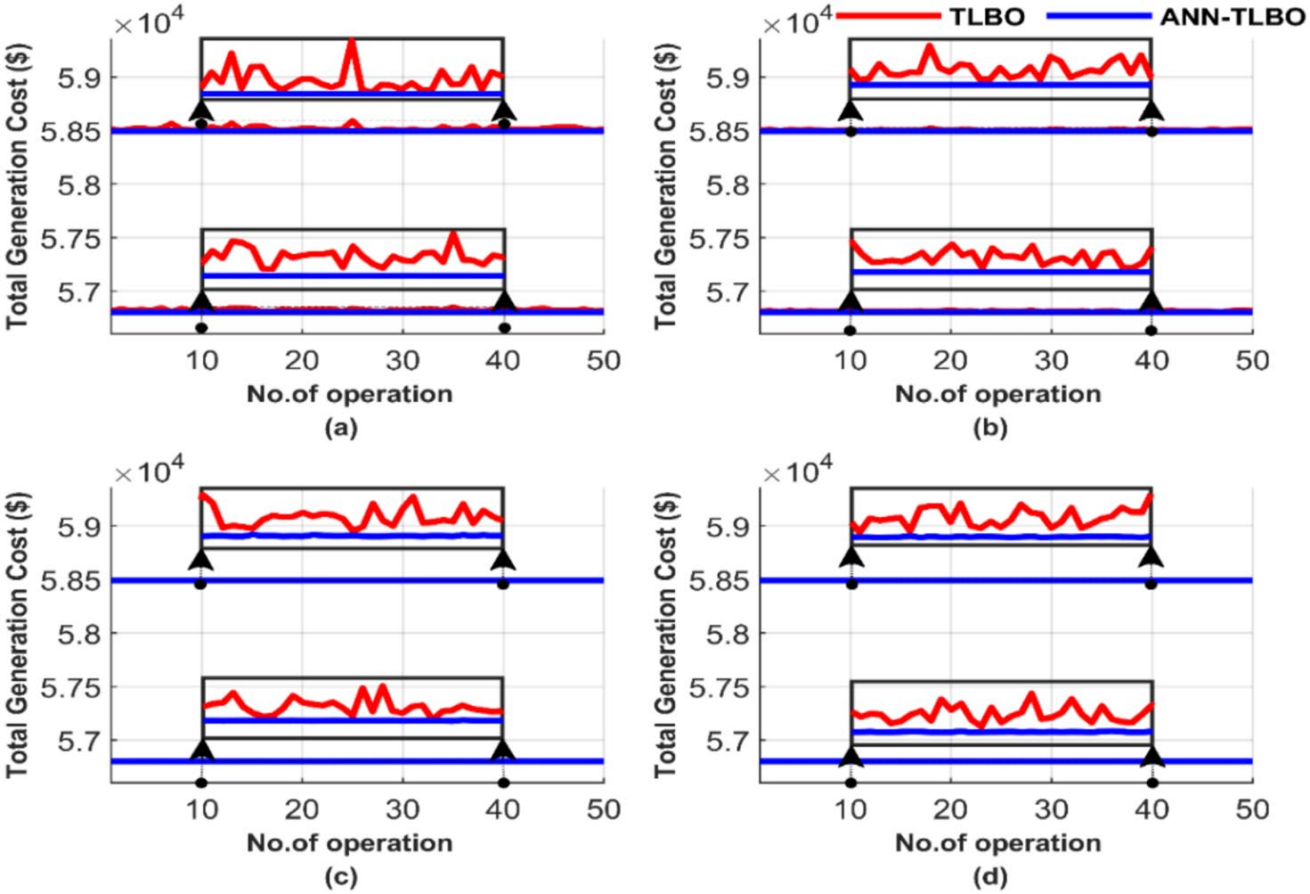
Comparison of cost curves over 50 runs: (**a**) 100 iterations, 25 populations, (**b**) 100 iterations, 50 populations, (**c**) 1000 iterations, 50 populations, (**d**) 1000 iterations, 100 populations.

Figure 21 provides a comparative analysis of the performance of the two methods across 50 runs, emphasizing iteration and population size differences. Figure 21 summarizes the results for four cases: (a) 100 iterations with 25 populations, (b) 100 iterations with 50 populations, (c) 1000 iterations with 50 populations, and (d) 1000 iterations with 100 populations.

The TLBO method performs better than GA and PSO in achieving the lowest cost and constancy, as shown in Fig. 21. However, the performance of TLBO declines when compared with the ANN-TLBO. The proposed AI-TLBO shows insensitivity to population and/or iteration variations. Meanwhile, the TLBO suffers from population and iteration dependency.

**Fig. 22:**
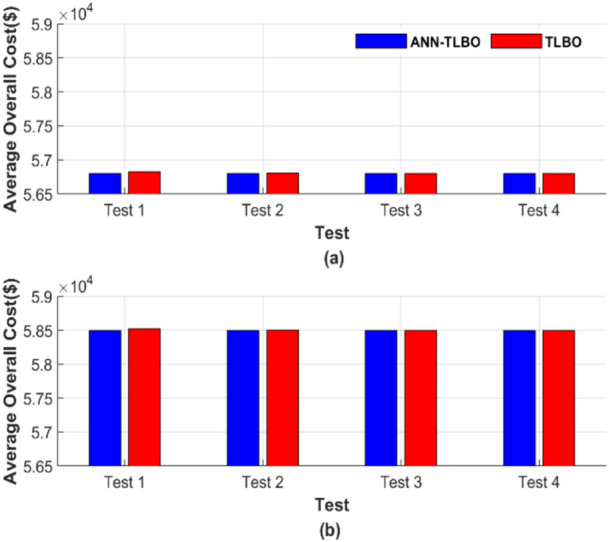
Average overall cost for ED problem: (**a**) without losses and (**b**) with losses.

Figure 22 presents the average total cost across 50 runs. Figure 22a depicts the average total generation cost under four operating conditions without accounting for losses, while Fig. 22b presents the corresponding results with losses included.

Figure 22 confirms the previously highlighted strength and reliability of ANN-TLBO. This method consistently achieved the lowest cost with exceptional constancy, regardless of potential variable changes. In Fig. 22a, the ANN-TLBO method and the TLBO method reached costs of 56,801.15 $ and 56,801.24 $, respectively. Similarly, in Fig. 22b, their corresponding costs were 58,491.41 $ and 58,491.51 $.

**Fig. 23:**
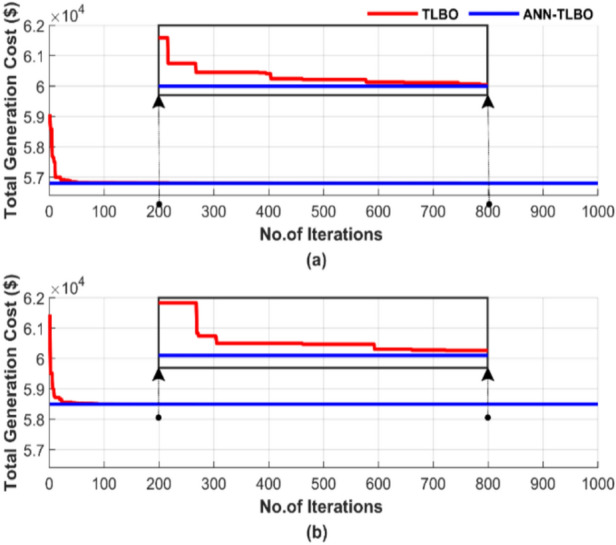
Cost convergence of two algorithms for ED problem over a single run with 1000 iterations and 50 populations: (**a**) without losses and (**b**) with losses.

Figure 23 illustrates the random behavior of the algorithms in a single run, using a population size of 50 and 1000 iterations.

Figure 23 illustrates that the ANN-TLBO method achieved a lower cost than the TLBO method. ANN-TLBO achieved a 0.00025% cost reduction in Fig. 23a and a 0.0002% reduction in Fig. 23b compared to TLBO. ANN-TLBO reached constancy more quickly. In contrast, the TLBO method required approximately 600 iterations to achieve constancy.

**Fig. 24:**
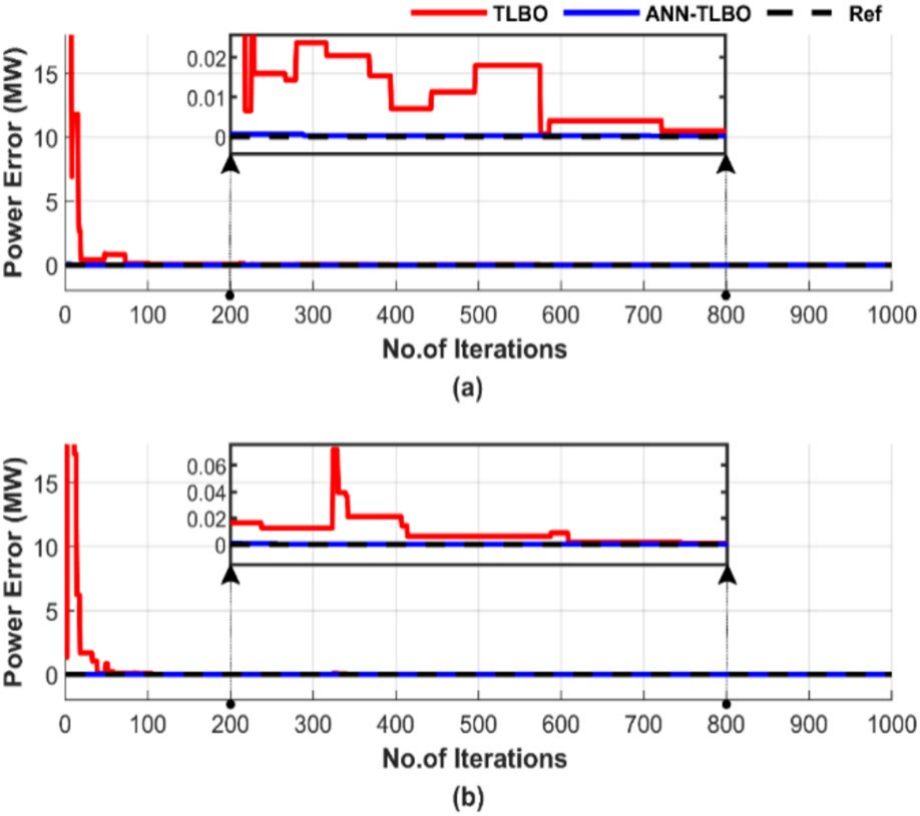
Power mismatch convergence of two algorithms for ED problem over a single run with 1000 iterations and 50 populations: (**a**) without losses and (**b**) with losses.

Figure 24 outlines the discrepancy between generated power and load demand during a single run, using a population size of 50 and 1000 iterations.

Figure 24 shows the performance difference between TLBO and ANN-TLBO methods in achieving power balance. The ANN-TLBO is characterized by rapid response and complete constancy, while TLBO needs time to stabilize approximately more than 600 iterations.

#### Scenario II-3: the load is 1000 MW, with and without losses

ANN-TLBO is tested and compared to TLBO for the 1000 MW load level. The evaluation was performed across four scenarios, each varying in iteration numbers and population size, to address a 1000 MW load requirement. Each scenario was run 50 times, factoring in population size and iteration variations. The results were taken from a simulation that once took into account the losses and once neglected them.

**Fig. 25:**
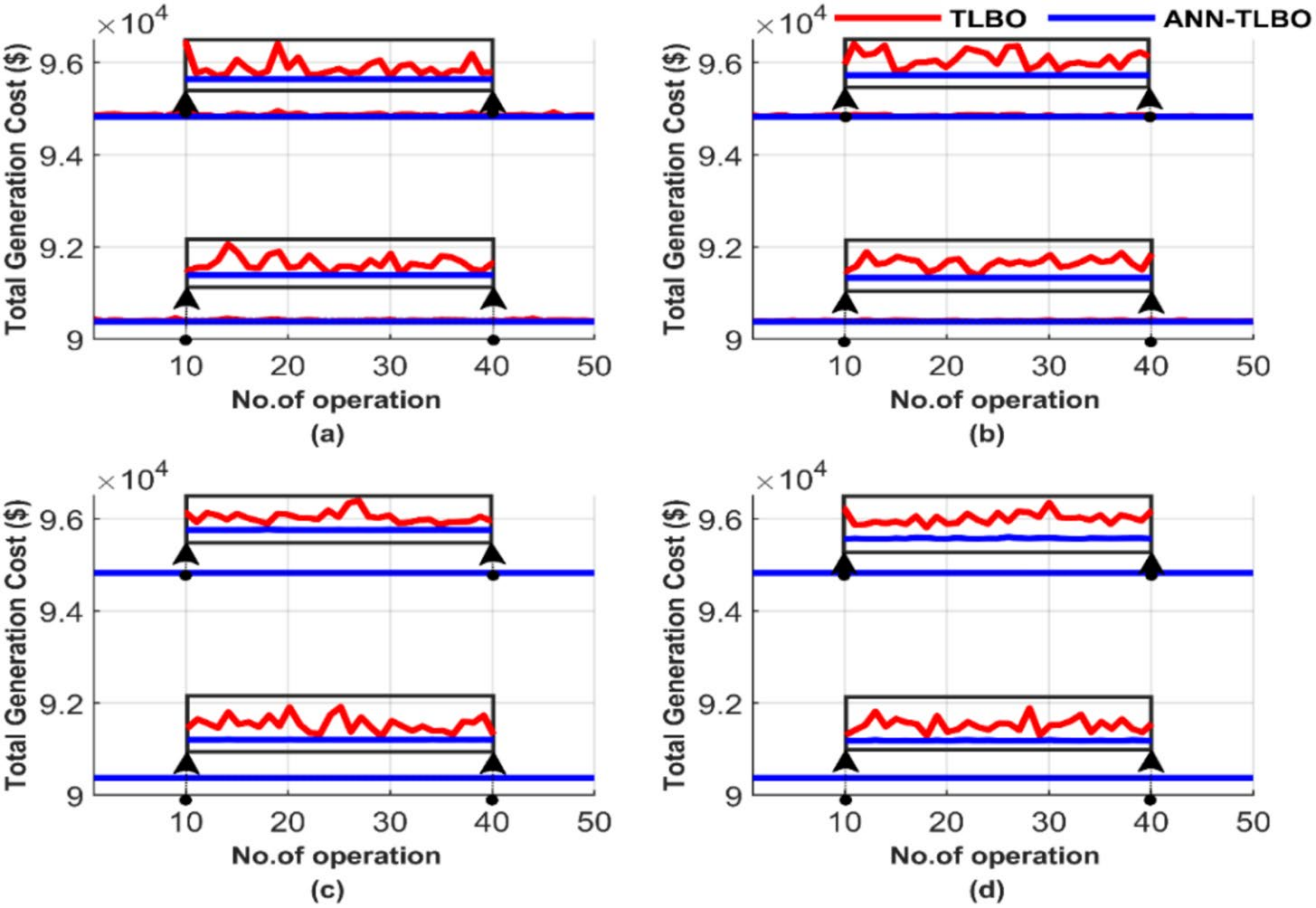
Comparison of cost curves over 50 runs: (**a**) 100 iterations, 25 populations, (**b**) 100 iterations, 50 populations, (**c**) 1000 iterations, 50 populations, (**d**) 1000 iterations, 100 populations.

Figure 25 illustrates the performance comparison between the two methods over 50 runs, focusing on the differences in iterations and population sizes. In Fig. 25, the results are shown for: (a) 100 iterations with 25 populations, (b) 100 iterations with 50 populations, (c) 1000 iterations with 50 populations, and (d) 1000 iterations with 100 populations.

Figure 25 shows that the AI-based optimizer, ANN-TLBO, performs better than the meta-heuristic optimizer regarding the global optimum value, which is the total generation cost. TLBO, however, has a higher quality solution than GA and PSO. This superiority is obvious from Figs. 5, 6, 7, 8, 9, 10, 11, 12, 13, 14, 15, 16, 17, 18, 19, 20, 21, 22, 23, 24 and 25. TLBO seems less affected by increasing population and/or iteration numbers than GA and PSO. Figure 25 shows that the TLBO solution varies across different runs in all subfigures presented, while ANN-TLBO is a constant irrespective of the run, iteration, and/or population.

**Fig. 26:**
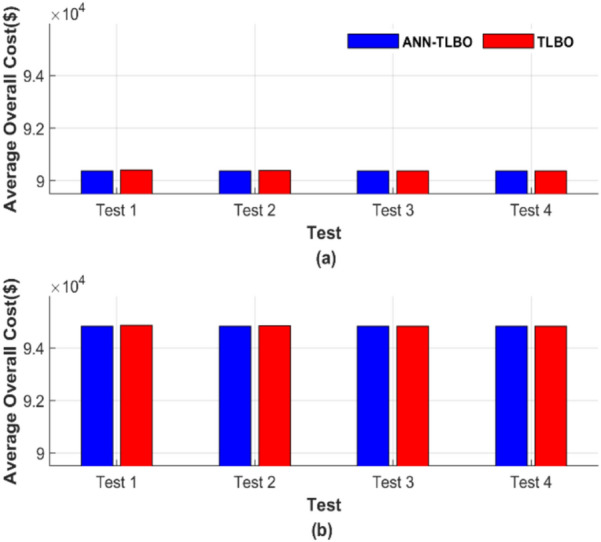
Average overall cost for ED problem: (**a**) without losses and (**b**) with losses.

Figure 26 presents the average total generation cost across various operating cases. Figure 26a shows the results without considering losses, while Fig. 26b includes the effects of losses.

From Fig. 26, the ANN-TLBO and TLBO methods achieved costs of 90,378.06 $ and 90,378.21 $, respectively, in Fig. 26a. Similarly, in Fig. 26b, their corresponding costs were 94,826.30 $ and 94,826.44 $, while the performance of both methods is comparable. ANN-TLBO method proves to be slightly less expensive.

**Fig. 27:**
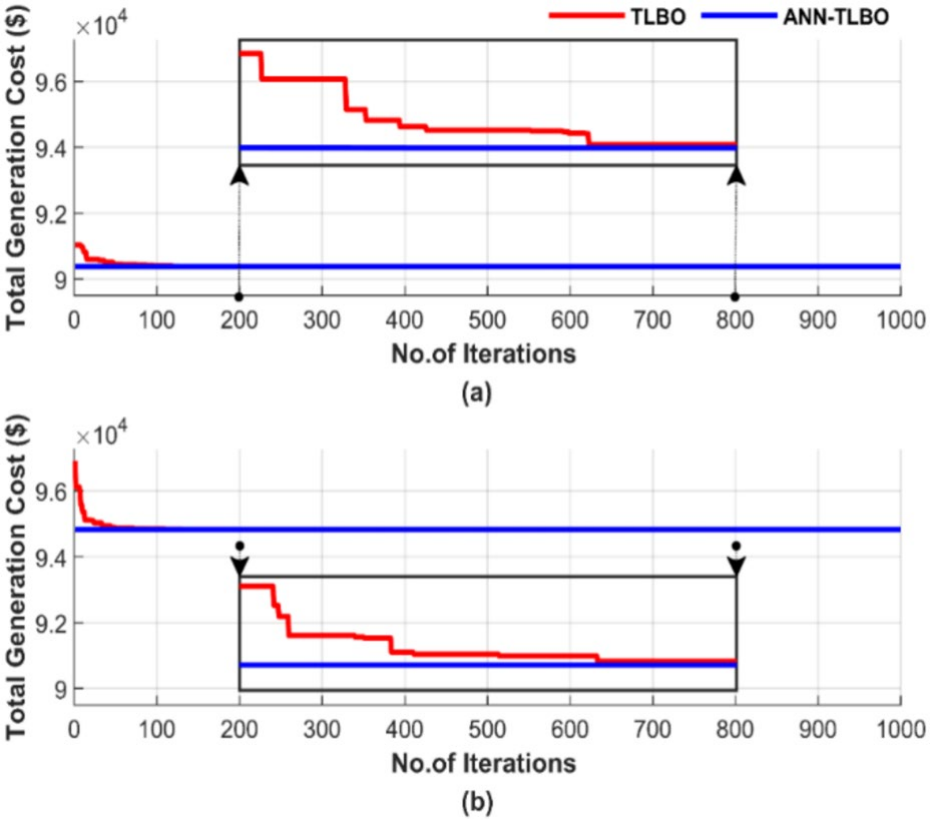
Cost convergence of two algorithms for ED problem over a single run with 1000 iterations and 50 populations: (**a**) without losses and (**b**) with losses.

Figure 27 illustrates the behavior of the algorithms during a randomly selected single run conducted with a population size of 50 and 1000 iterations. Figure 27a displays the results without accounting for losses, while Fig. 27b includes the impact of losses.

Figure 27 confirms the advantage of ANN-TLBO in achieving the lowest cost stably and rapidly compared to the TLBO method, where ANN-TLBO achieved a 0.00028% cost reduction compared to TLBO in Fig. 27a and 0.00026% reduction compared to TLBO in Fig. 27b. TLBO requires more than 600 iterations to approach the performance of ANN-TLBO, while the ANN-TLBO was settled immediately.

**Fig. 28:**
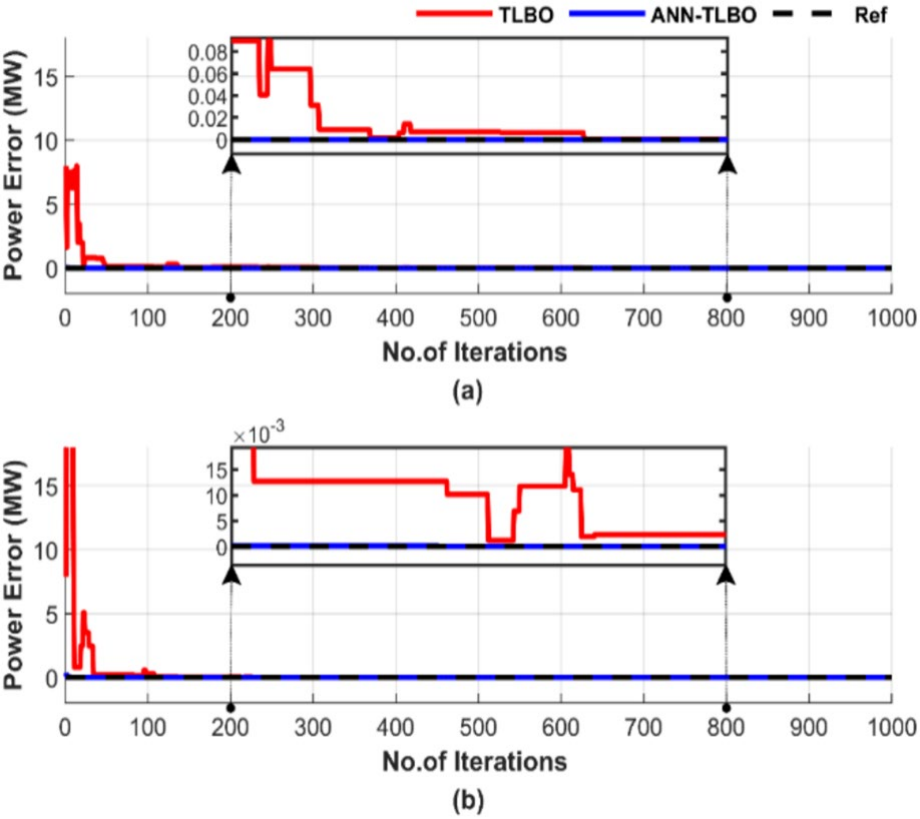
Power mismatch convergence of two algorithms for ED problem over a single run with 1000 iterations and 50 populations: (**a**) without losses and (**b**) with losses.

Figure 28 highlights the difference between generated power and load demand during a single run, using a population size of 50 and 1000 iterations. Figure 28a displays the results without accounting for losses, while Fig. 28b includes the impact of losses.

Figure 28 demonstrates that both methods can match generated power to load. In Fig. 28a, ANN-TLBO stabilizes much faster, unlike TLBO, which requires more than 600 iterations to achieve constancy. However, Fig. 28b shows the accuracy and efficiency of ANN-TLBO in achieving power balance.

### Test case 4: the performance of the ANN-AGTO method

#### Scenario I-4: the load is 700 MW, with and without losses

AGTO was used to test the proposed technique. The following figures show the results from AGTO and AI-AGTO in identifying the most promising optimizer. Moreover, to continue testing for the proposed hybrid AI-meta-heuristic optimizer. The evaluation covers four scenarios with different iterations and population sizes to meet a load demand of 700 MW. Each case was tested 50 times, considering variations in population size and iteration number.

**Fig. 29:**
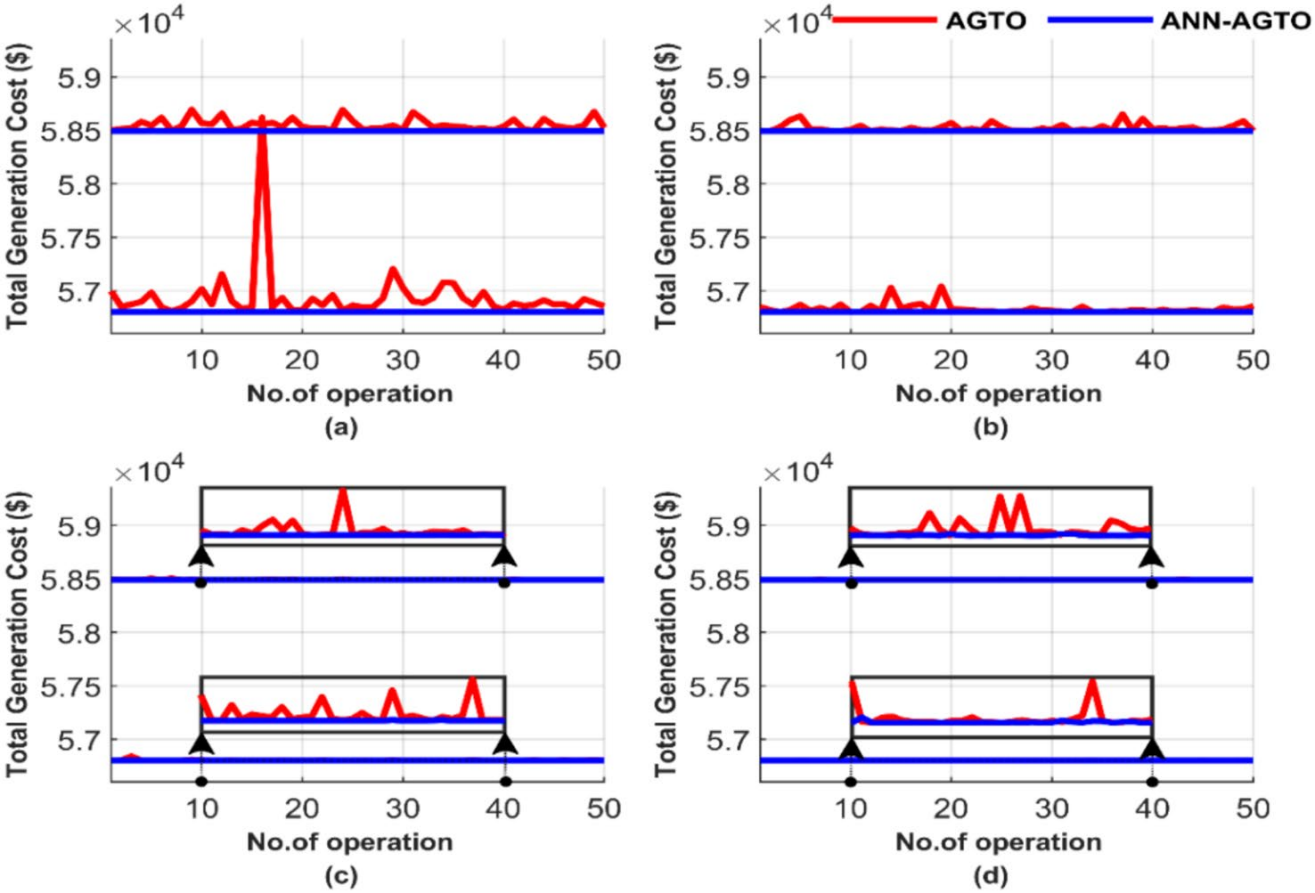
Comparison of cost curves over 50 runs: (**a**) 100 iterations, 25 populations, (**b**) 100 iterations, 50 populations, (**c**) 1000 iterations, 50 populations, (**d**) 1000 iterations, 100 populations.

Figure 29 compares the performance of two methods over these 50 runs, emphasizing iteration and population size differences. The data in Fig. 29 highlights four scenarios: (a) 100 iterations paired with a population size of 25, (b) 100 iterations paired with a population size of 50, (c) 1000 iterations paired with a population size of 50, and (d) 1000 iterations paired with a population size of 100.

ANN-AGTO shows in Fig. 29 constancy and effectiveness in achieving the lowest cost regardless of operating conditions, unlike the traditional method AGTO, which is characterized by high sensitivity to changes in population size and the number of iterations. The performance of AGTO improves notably as the population size and number of iterations increase. In contrast, the performance of ANN-AGTO remains nearly unchanged, regardless of variations in population size or iteration number.

**Fig. 30:**
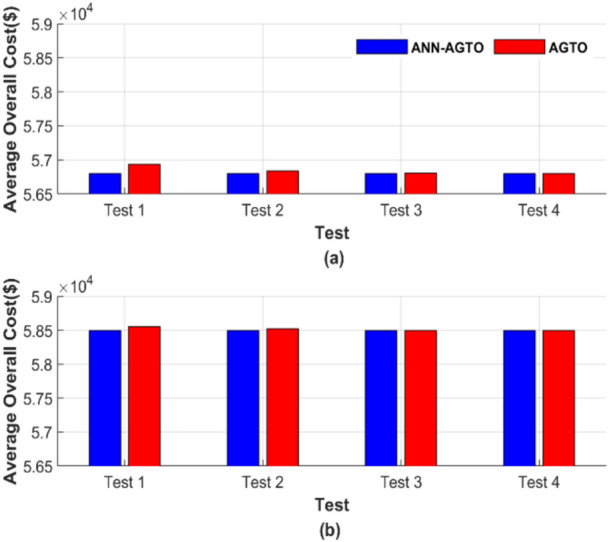
Average overall cost for ED problem: (**a**) without losses and (**b**) with losses.

The average total generation cost for each test was calculated, and the results are presented in Fig. 30. Figure 30 comprises two subfigures: Fig. 30a depicts the average total generation cost for the four operating conditions without losses, and Fig. 30b illustrates the results when losses are considered.

Figure 30 depicts that ANN-AGTO consistently outperformed AGTO in achieving the lowest costs across all scenarios. Specifically, AGTO incurred 56,801.24 $ and 58,491.52 $ for Fig. 30a and b, respectively, whereas ANN-AGTO achieved lower costs of 56,801.16 $ and 58,491.42 $ for the corresponding subfigures.

**Fig. 31:**
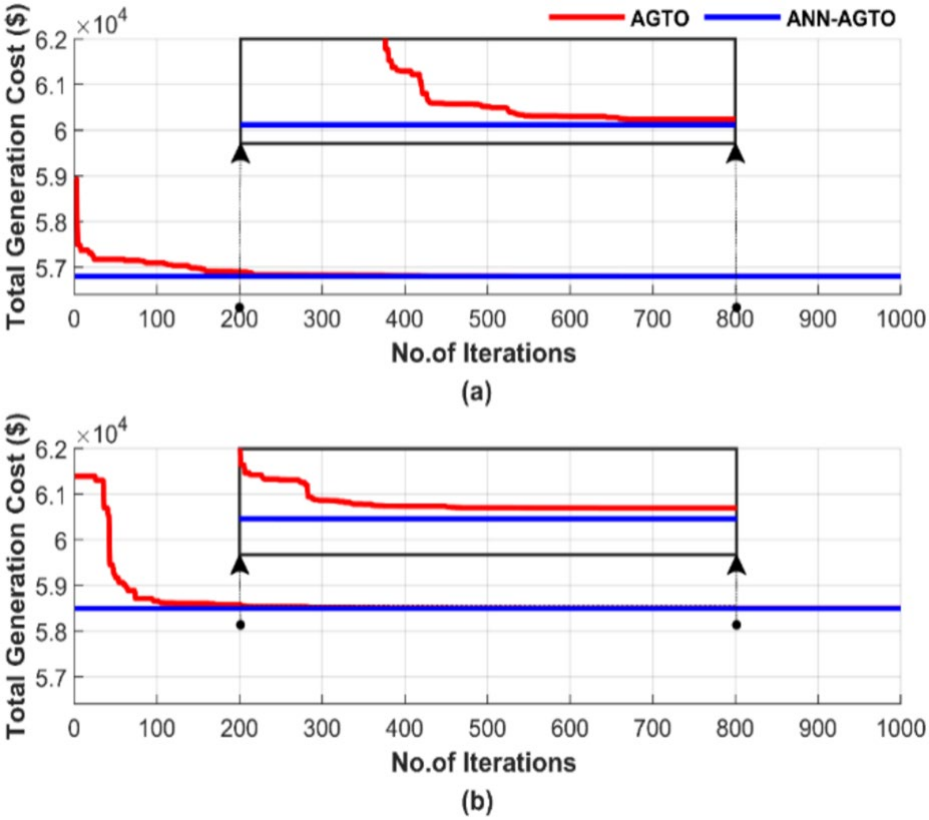
Cost convergence of two algorithms for ED problem over a single run with 1000 iterations and 50 populations: (**a**) without losses and (**b**) with losses.

Figure 31 demonstrates the convergence of the algorithms in a single run for the four scenarios, using a population size of 50 and 1000 iterations. Figure 31 contains two subfigures: Fig. 31a shows the total generation cost for the operating conditions without accounting for losses, while Fig. 31b presents the results when losses are considered.

Figure 31 demonstrates the superiority of ANN-AGTO in achieving the lowest cost constancy compared to the AGTO method. ANN-AGTO method achieves a 0.003% cost reduction in Fig. 31a and a 0.0002% reduction in Fig. 31b compared to AGTO. PSO-AGTO exhibited consistent performance from the outset, whereas AGTO required over 500 iterations to reach a comparable level of effectiveness.

**Fig. 32:**
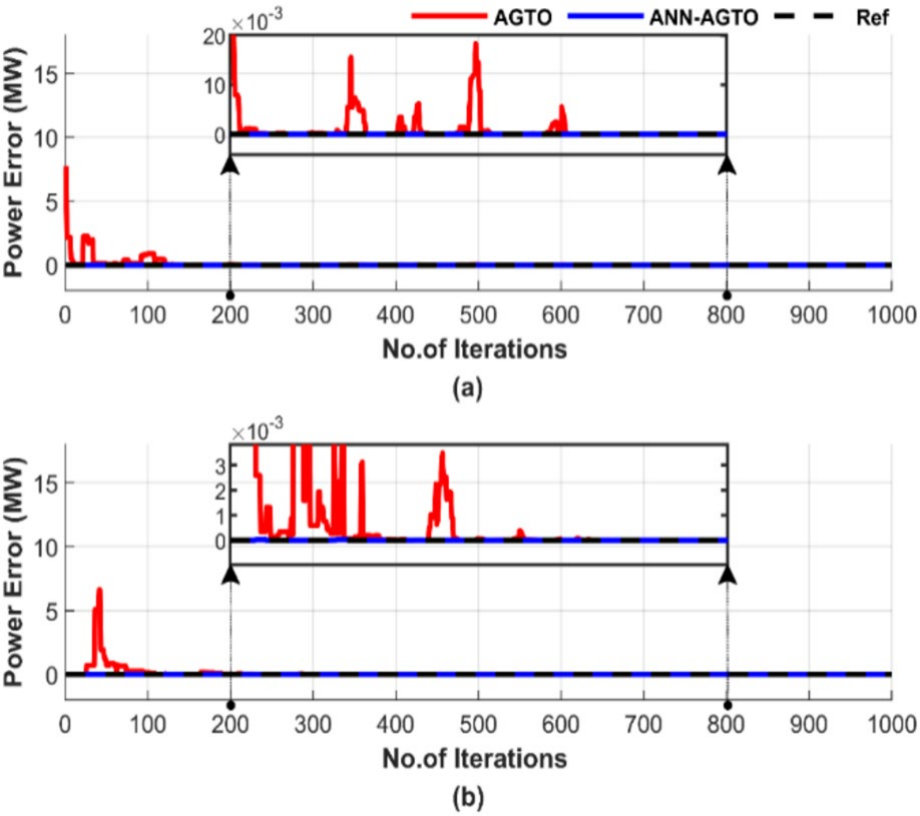
Power mismatch convergence of two algorithms for ED problem over a single run with 1000 iterations and 50 populations: (**a**) without losses and (**b**) with losses.

Figure 32 illustrates the difference between generated power and load demand. Figure 32 features two subfigures: Fig. 32a displays the power mismatch for the operating conditions without including losses, and Fig. 32b illustrates the results with losses factored in.

Figure 32 shows that while both methods can balance power output with load, ANN-AGTO stabilizes much more quickly than AGTO, which requires more than 500 iterations to reach constancy, either in Fig. 32a or b.

#### Scenario II-4: the load is 1000 MW, with and without losses

The results from AGTO and ANN-AGTO for 1000 MW were illustrated here to continue testing the targeted AI-GPSed optimizer. The test was conducted across four scenarios with different iteration counts and population sizes. Each scenario was executed 50 times, accounting for population size and iteration number variations.

**Fig. 33:**
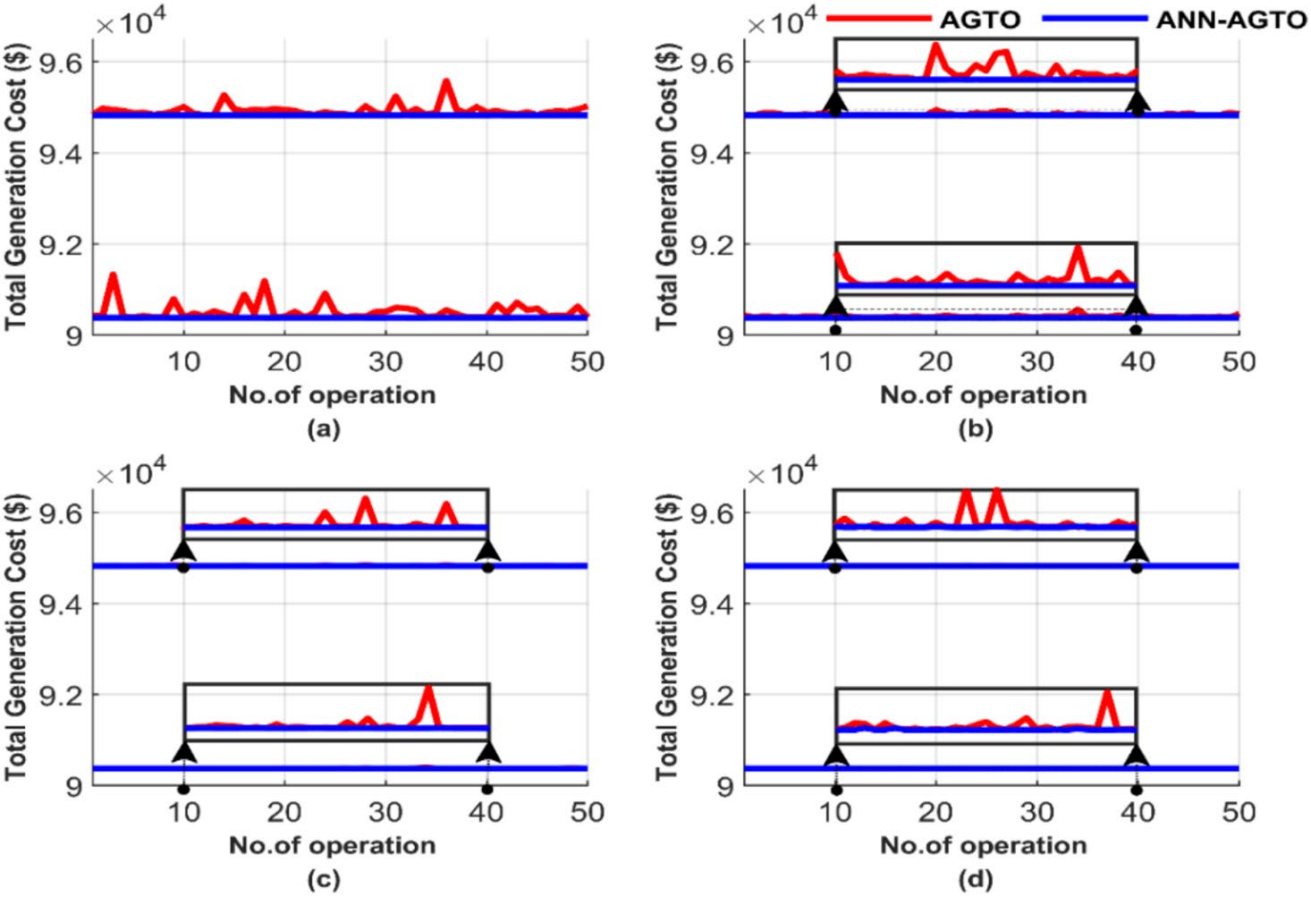
Comparison of cost curves over 50 runs: (**a**) 100 iterations, 25 populations, (**b**) 100 iterations, 50 populations, (**c**) 1000 iterations, 50 populations, (**d**) 1000 iterations, 100 populations.

Figure 33 presents a performance comparison between the two methods over 50 runs, highlighting differences in iterations and population size. Figure 33 depicts the outcomes for the following cases: (a) 100 iterations using 25 populations, (b) 100 iterations using 50 populations, (c) 1000 iterations using 50 populations, and (d) 1000 iterations using 100 populations.

In Fig. 33, with the same population size and iteration number, AGTO shows inconsistent and unstable results, and ANN-AGTO produces stable and consistent lower values. Comparing the four scenarios, it is clear that AGTO is highly sensitive to changes in population size and iteration numbers, as its performance fluctuates significantly. In contrast, ANN-AGTO maintains near-constant performance regardless of these changes. This constancy is attributed to the guided approach used by ANN, which leads it to the quickest and most stable solution path, unlike the traditional method that starts with random values.

**Fig. 34:**
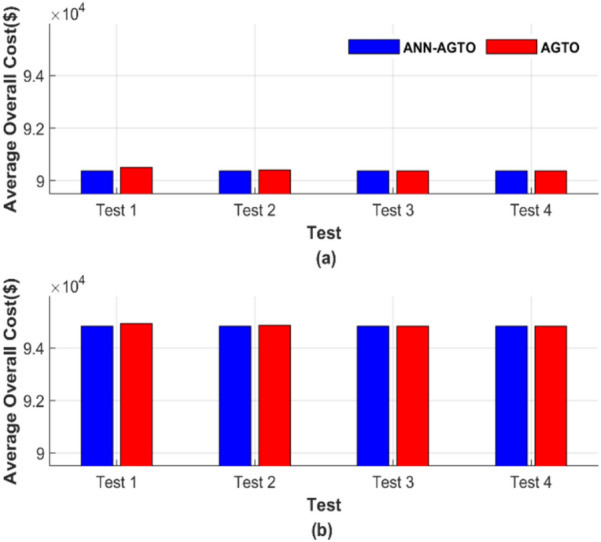
Average overall cost for ED problem: (**a**) without losses and (**b**) with losses.

Figure 34 shows the graphical representation of the average cost for each scenario. Figure 34 consists of two subfigures: Fig. 34a depicts the average total generation cost for the four operating conditions without accounting for losses. In contrast, Fig. 34b illustrates the results when losses are included.

Figure 34 graphically confirms the constancy of ANN-AGTO, in contrast to the sensitivity of AGTO to population size and iteration count. AGTO recorded 90,378.15 $ and 94,826.44 $ for Fig. 34a and b, respectively, while ANN-AGTO achieved reduced costs of 90,378.06 $ and 94,826.30 $ for the same subfigures.

**Fig. 35:**
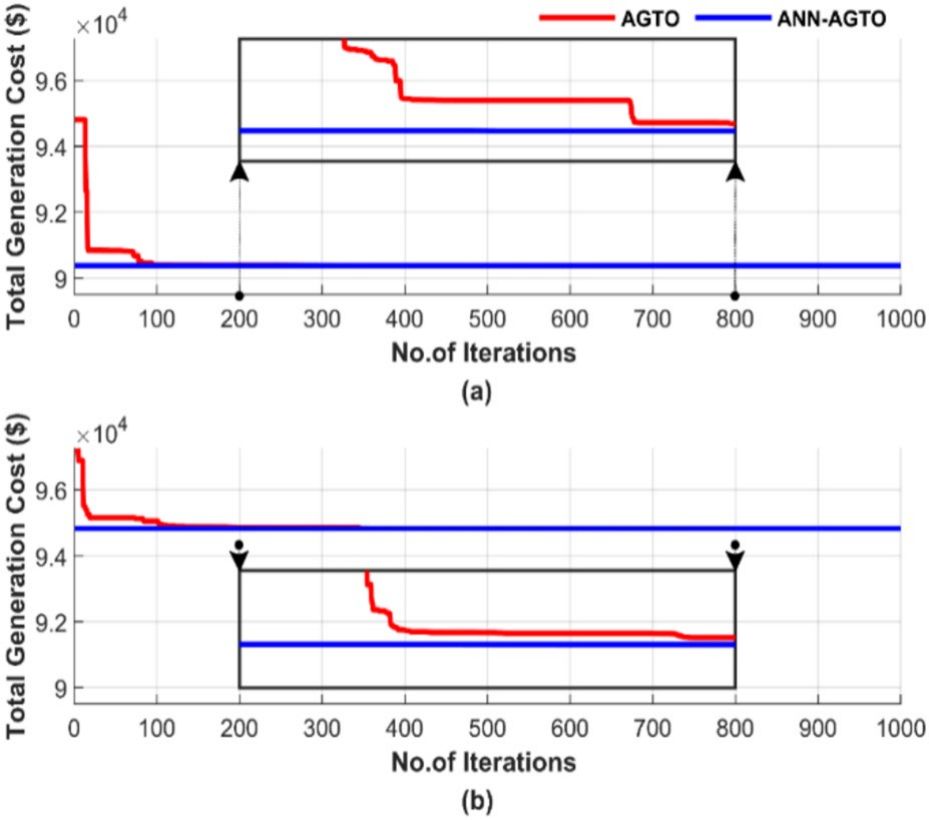
Cost convergence of two algorithms for ED problem over a single run with 1000 iterations and 50 populations: (**a**) without losses and (**b**) with losses.

Figure 35 illustrates the random behavior of the algorithms in a single run for the four scenarios, using a population size of 50 and 1000 iterations. Figure 35 includes two subfigures: Fig. 35a shows the total generation cost without losses, while Fig. 35b displays the results with losses taken into account.

Figure 35 shows that the ANN-AGTO excels in quickly and reliably achieving the lowest cost, whereas the AGTO method requires more than 700 iterations to approach the performance achieved by ANN-AGTO. ANN-AGTO method reduces costs by 0.0012% in Fig. 35a and 0.0014% in Fig. 35b compared to AGTO. These results highlight the effectiveness of the ANN-AGTO method in achieving optimal solutions more efficiently than the AGTO method.

**Fig. 36:**
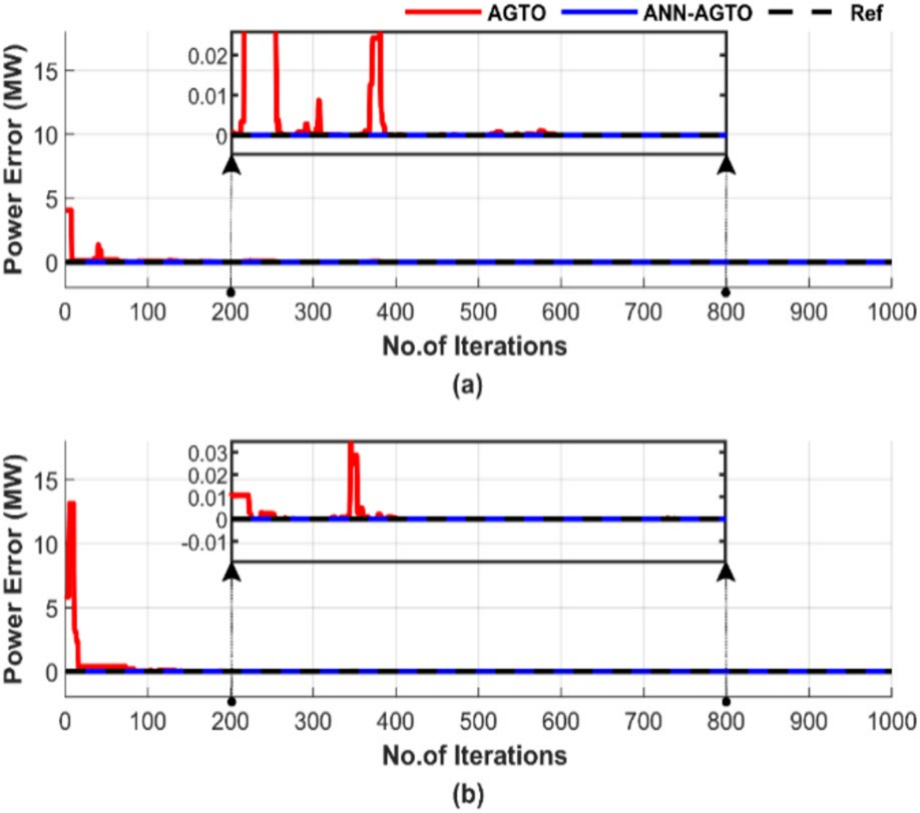
Power mismatch convergence of two algorithms for ED problem over a single run with 1000 iterations and 50 populations: (**a**) without losses and (**b**) with losses.

Figure 36 displays the discrepancy between the generated power and the load demand. Figure 36a displays the results for conditions without including losses, and Fig. 36b illustrates the results with losses.

Figure 36 demonstrates that both methods can balance power generation, but ANN-AGTO stabilizes much faster. In Fig. 36a, ANN-AGTO outperforms AGTO by stabilizing more quickly. AGTO stabilizes after 600 iterations. Additionally, Fig. 36b highlights that AGTO stabilizes after 400 iterations, while ANN-AGTO is stable from the beginning.

## Comparison and statistical performance

The results shown in Figs. 5, 6, 7, 8, 9, 10, 11, 12, 13, 14, 15, 16, 17, 18, 19, 20, 21, 22, 23, 24, 25, 26, 27, 28, 29, 30, 31, 32, 33, 34, 35 and 36 demonstrate the effectiveness and robustness of the proposed AI-GPSed optimizer compared to the meta-heuristic optimizer alone. The results indicate that the proposed AI-GPSed optimizer consistently converges to the global optimum with the fewest iterations and populations/particles irrespective of the parameters and/or operating conditions.

To ensure a more comprehensive and fair assessment of the proposed AI-GPSed optimizer method, Tables 3, 4, 5 and 6 compare statistical performance measures and computational efficiency. These tables include ablation studies in which the ANN component was removed from the hybrid method to facilitate a direct comparison between the AI-GPSed optimizer and its individual counterparts, GA, PSO, TLBO, and AGTO methods under various load levels and operating conditions at 1000 iterations, and 50 populations. Table 3 reports the standard deviation over 50 runs for the AI-GPSed optimizer and the candidate optimizers. Table 4 presents the *p*-value from the Wilcoxon rank-sum test at a 5% significance level based on 50 runs. Table 5 demonstrates the confidence interval analysis over 50 runs for the AI-GPSed optimizer and the candidate optimizers. Table 6 depicts the computational efficiency comparison under the same experimental settings.

**Table 3:** Standard deviation test results of the AI-GPSed optimizer compared to the four traditional methods over 50 runs.

Load (MW)	Losses	ANN-GA	GA	ANN-PSO	PSO	ANN-TLBO	TLBO	ANN-AGTO	AGTO
700	Without	0.008406122	77.53660728	0.00603261	0.392228425	0.002779971	0.069864714	0.019156636	5.712028154
700	With	0.008526658	58.68352507	0.005043479	0.415736353	0.003014938	0.065972187	0.015604	2.466363396
1000	Without	0.00971797	80.03744099	0.006956166	0.70528246	0.002242319	0.126883354	0.010885655	2.640581902
1000	With	0.009355813	77.49626751	0.006108874	0.435187508	0.002529071	0.129731888	0.016911248	2.276456326

**Table 4:** The* p*-value of the Wilcoxon rank-sum test at a 5% significance level for the AI-GPSed optimizer compared to the four traditional methods over 50 runs.

Load (MW)	Losses	ANN-GA versus GA		ANN-PSO versus PSO		ANN-TLBO versus TLBO		ANN-AGTO versus AGTO	
		*p*-value	R	*p*-value	R	*p*-value	R	*p*-value	R
700	Without	1.77636E-15	+	1.77636E-15	+	1.77636E-15	+	5.32907E-15	+
700	With	1.77636E-15	+	1.77636E-15	+	1.77636E-15	+	3.55271E-15	+
1000	Without	1.77636E-15	+	1.77636E-15	+	1.77636E-15	+	1.77636E-15	+
1000	With	1.77636E-15	+	1.77636E-15	+	1.77636E-15	+	1.77636E-15	+

**Table 5:** 95% Confidence Interval test of the AI-GPSed optimizer compared to the four traditional methods over 50 runs.

Load (MW)	Losses	ANN-GA	GA	ANN-PSO	PSO	ANN-TLBO	TLBO	ANN-AGTO	AGTO
700	Without	[56801.16, 56,801.17]	[56849.38, 56,893.45]	[56801.16, 56,801.17]	[56801.53, 56,801.76]	[56801.16, 56,801.17]	[56801.53, 56,801.76]	[56801.16, 56,801.17]	[56801.21, 56,804.46]
700	With	[58491.42, 58,491.43]	[58529.27, 58,562.63]	[58491.42, 58,491.43]	[58491.79, 58,492.01]	[58491.42, 58,491.43]	[58491.79, 58,492.01]	[58491.42, 58,491.43]	[58491.83, 58,493.24]
1000	Without	[90378.07, 90,378.08]	[90441.11, 90,486.60]	[90378.06, 90,378.07]	[90378.67, 90,379.07]	[90378.06, 90,378.07]	[90378.67, 90,379.07]	[90378.06, 90,378.07]	[90378.40, 90,379.90]
1000	With	[94826.31, 94,826.32]	[94899.78, 94,943.83]	[94826.30, 94,826.31]	[94826.81, 94,827.05]	[94826.30, 94,826.31]	[94826.81, 94,827.05]	[94826.31, 94,826.32]	[94826.93, 94,828.23]

**Table 6:** Computational efficiency results of the AI-GPSed optimizer compared to the four traditional methods over 50 runs.

Load (MW)	Losses	ANN-GA	GA	ANN-PSO	PSO	ANN-TLBO	TLBO	ANN-AGTO	AGTO
700	Without	1.40	1.77	1.39	1.56	1.49	1.45	1.53	1.55
700	With	1.59	2.23	1.78	1.88	2.30	2.20	2.18	2.07
1000	Without	1.38	1.69	1.49	1.51	1.51	1.46	1.54	1.51
1000	With	1.88	2.07	1.93	1.97	2.13	2.18	2.45	2.20

Table 3 demonstrates that the AI-GPSed optimizer exhibits a significantly lower standard deviation than traditional methods, which show much higher variability across all operating conditions and scenarios. This superiority further confirms the capability previously illustrated in Figs. 5, 6, 7, 8, 9, 10, 11, 12, 13, 14, 15, 16, 17, 18, 19, 20, 21, 22, 23, 24, 25, 26, 27, 28, 29, 30, 31, 32, 33, 34, 35 and 36, highlighting the consistency of the AI-GPSed optimizer in achieving the lowest cost, in contrast to the fluctuations and instability observed in traditional methods.

Table 4 confirms the consistency of the AI-GPSed optimizer in achieving the lowest cost, as it demonstrates a statistically significant difference between the two methods across all operating conditions and scenarios. This consistency indicates that the AI-GPSed optimizer outperformed traditional methods in terms of cost minimization. It is also noted that the *p*-values are equal in most cases, which can be attributed to the similarity in the relative performance of the AI-GPSed optimizer method.

Table 5 further confirms the consistency and efficiency of the proposed AI-GPSed optimizer. The confidence interval width of 0.01 reported for the AI-GPSed optimizer indicates a highly precise estimate, likely resulting from low variability within the sample. In contrast, traditional methods exhibit a significantly wider confidence interval, reflecting greater uncertainty due to higher variability. Such wider intervals necessitate caution when interpreting results, whereas the narrower intervals associated with AI-GPSed optimizers offer more reliable and robust optimization.

Table 6 shows that the AI-GPSed optimizer consistently requires less computational time than traditional methods, confirming the fast convergence observed in the preceding figures—except for a few operating scenarios. However, this can be mitigated by reducing the number of iterations and population size, as the AI-GPSed optimizer maintains stable and consistent performance regardless of the number of runs, iterations, or population size. Some disturbances in execution times may be caused by the operating condition of the equipment, its age, and non-ideal operating conditions such as temperature. It is worth mentioning that the equipment where the computation is carried out has the specifications shown in Table 7.

**Table 7:** Computer system specifications.

Component	Specification
Processor	Intel(R) Core(TM) i7-8565U CPU @ 1.80 GHz 1.99 GHz
Operating System	Windows 10
RAM	16.0 GB
GPU	NVIDIA GeForce MX130

Tables 3, 4, 5 and 6 confirm that the ablation of the ANN phase has a notable impact on all outcomes, affecting both statistical performance represented by achieving the lowest cost consistently and execution time. The proposed AI-GPSed optimizer demonstrates both efficiency and reliability across all cases and under various operating conditions, consistently achieving lower costs in less time than traditional methods.

## Conclusion and future works

An innovative AI-GPSed optimizer is proposed in this article. Usually, the optimization techniques use random initial solutions, which deteriorate their performance and affect the quality of the produced solution. The proposed optimizer uses AI to find the most suitable initial solution. Thus, the proposed optimizer consists of two phases. Phase 1, where the AI determines the initial solution. In the second phase, a meta-heuristic optimizer determines the global optimum solution. Four different meta-heuristic optimizers: GA, PSO, TLBO, and AGTO, were utilized in the second phase to validate the proposed optimizer. The proposed optimizer has been applied for efficient and stable optimization of power generation from units in ED on a standard IEEE 30-bus system with six generating units. The results, Figs. 5, 6, 7, 8, 9, 10, 11, 12, 13, 14, 15, 16, 17, 18, 19, 20, 21, 22, 23, 24, 25, 26, 27, 28, 29, 30, 31, 32, 33, 34, 35 and 36 and Tables 3, 4, 5 and 6 clearly demonstrate the strength and robustness of the proposed algorithm, highlighting its ability to achieve the lowest cost with full constancy and significantly faster convergence compared to traditional methods. The key conclusions can be summarized as follows:

- The proposed mixing of AI and optimization algorithms has performed better than traditional methods.
- The proposed method resulted in lower costs and improved convergence.
- In terms of constancy, the proposed technique is not only more stable but also faster.
- Regarding accuracy, the method has demonstrated precise performance, as reflected in the quality of generated power and the minimal power discrepancies.
- Regarding sensitivity, the proposed technique is significantly less sensitive to variables like population size or iteration number compared to traditional methods.

All of the above proves that AI has enhanced the performance of the optimization methods by enabling faster convergence to more accurate solutions. The ED problem of the 30 IEEE Bus system is used to evaluate the proposed AI-GPSed optimizer. However, the proposed optimizer is not limited to this application and can be extended to address a wide range of engineering, economic, and scientific problems.

It is worth mentioning that the performance of the proposed optimization-driven AI technique in the case study is quite satisfactory and promising. However, applying the proposed hybrid method in other applications and/or ED with different operating scenarios could result in identifying some challenges.

In following studies, the proposed AI-GPSed optimizer would be evaluated on larger ED systems with more complex objective functions and constraints. AI-GPSed optimizers will also be assessed for ED problems involving real power system configurations and various operating scenarios. Adopting the model presented in this study for broader applications across different problem domains would be under consideration in future research. The proposed AI-GPSed optimizer would also be examined in dynamic economic dispatch and systems incorporating renewable energy sources. Furthermore, future research may extend its use to other fields, such as smart grid energy management, robotic surgery, and industrial robotics, and explore its integration with various optimization techniques to evaluate its effectiveness further.

## Data Availability

All data generated or analyzed during this study are included in this published article.
